# Gaps in AI-Driven Pharmacokinetic Property Prediction for Early Drug Development: A Scoping Review

**DOI:** 10.1021/acs.jcim.6c01331

**Published:** 2026-08-01

**Authors:** Lucille Tomin, Vida Bodaghi-Namileh, Diane G. Schwartz, Ram Samudrala, Zackary Falls

**Affiliations:** Department of Biomedical Informatics, Jacobs School of Medicine and Biomedical Sciences, 12291 University at Buffalo, Buffalo, New York 14214, United States

**Keywords:** pharmacokinetics, PK properties, ADME, machine learning, artificial intelligence, computational prediction, drug discovery, drug development, *in silico* method

## Abstract

Machine learning applications in preclinical drug development have been focused on automated covariate selection in pharmacometric modeling and high-throughput screening processes early in drug discovery. While inherent drug property prediction has made significant improvements in the past decade, fusing early target-based drug discovery methods to preclinical stage pharmacokinetic (PK) property predictions has been limited. This scoping review investigates the current state of PK property prediction of small molecules in drug discovery using machine learning methods and a combination of machine learning and mechanistic models. We identified major obstacles hindering the development of superior prediction models for small molecule behavior in biological systems. These encompass data accessibility, quantity, and quality, architectural constraints such as poor interpretability and model inherent assumptions, and the lack of robust evaluation and uncertainty assessment methods. To mitigate data-related constraints, we advocate for the use of collaborative federated learning frameworks. Furthermore, we propose leveraging the pattern recognition capabilities of deep learning models in conjunction with the biological interpretability provided by mechanistic approaches to strike an optimal balance between accuracy and biological explainability guided by the intended application of the prediction model. Addressing these limitations will advance reliable modeling pipelines and enable effective extrapolation to novel chemical space, additional species, and emerging drug development scenarios.

## Graphical abstract

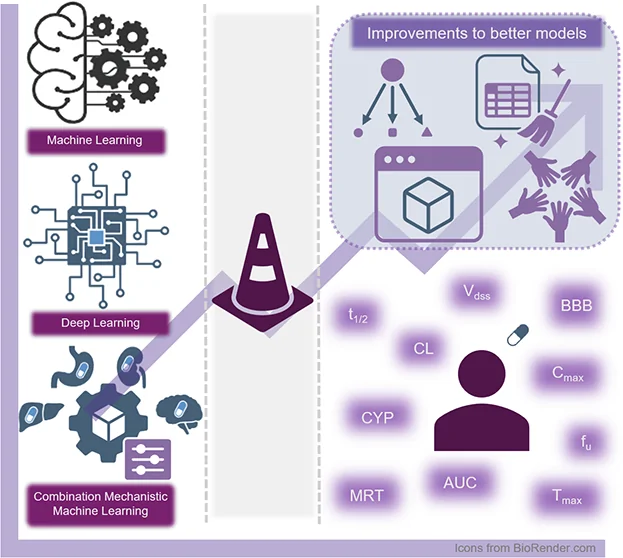

## 1 Introduction

Recent advances in computational methods and modeling accuracy have accelerated the therapeutic research space. However, drug discovery slowed in recent years as expressed by Eroom’s Law (inverse of Moore’s law) describing the phenomenon of increasing resources and time required for the development and approval of new drug candidates, resulting in a dramatic deceleration in novel therapeutic discovery. Advancements in artificial intelligence (AI), particularly in target-based small molecule discovery, gained significant traction in the bioinformatics and computational pharmaceutics space, as they greatly reduced the time and resources needed to bring new drugs to market.^1^

Target-based drug discovery begins by identifying a disease relevant molecular target (typically a protein). Then, a chemical library is screened for compound-target interactions identifying potential drug hits. The pipeline proceeds to further analysis of the identified hits and lead optimization before moving to preclinical studies and preparations for clinical trials (CTs)^1,2^ (Figure 1). Computational techniques for target affinity evaluation and compound screening implement chemical molecular descriptors to represent small molecules in a computer readable format. These structural representations range from zero to three-dimensional descriptors for feature-based learning. Common zero and one-dimensional features are chemical formula, molecular weight, number of rings, functional groups, or fragments. For instance, simplified molecular input line entry system (SMILES) strings are one-dimensional molecular descriptors. Two dimensional representations include contextual information about the molecule through molecular fingerprinting techniques, while three-dimensional spatial characterization techniques include the surface area, volume, and steric properties of the molecule through geometrical or pharmacophore fingerprints.^3^ Representing a library of compounds using these techniques allows for the implementation of computational high throughput screening methods rapidly identifying lead compounds for further investigation at early stages of drug discovery.^1,2^

**Figure 1:**
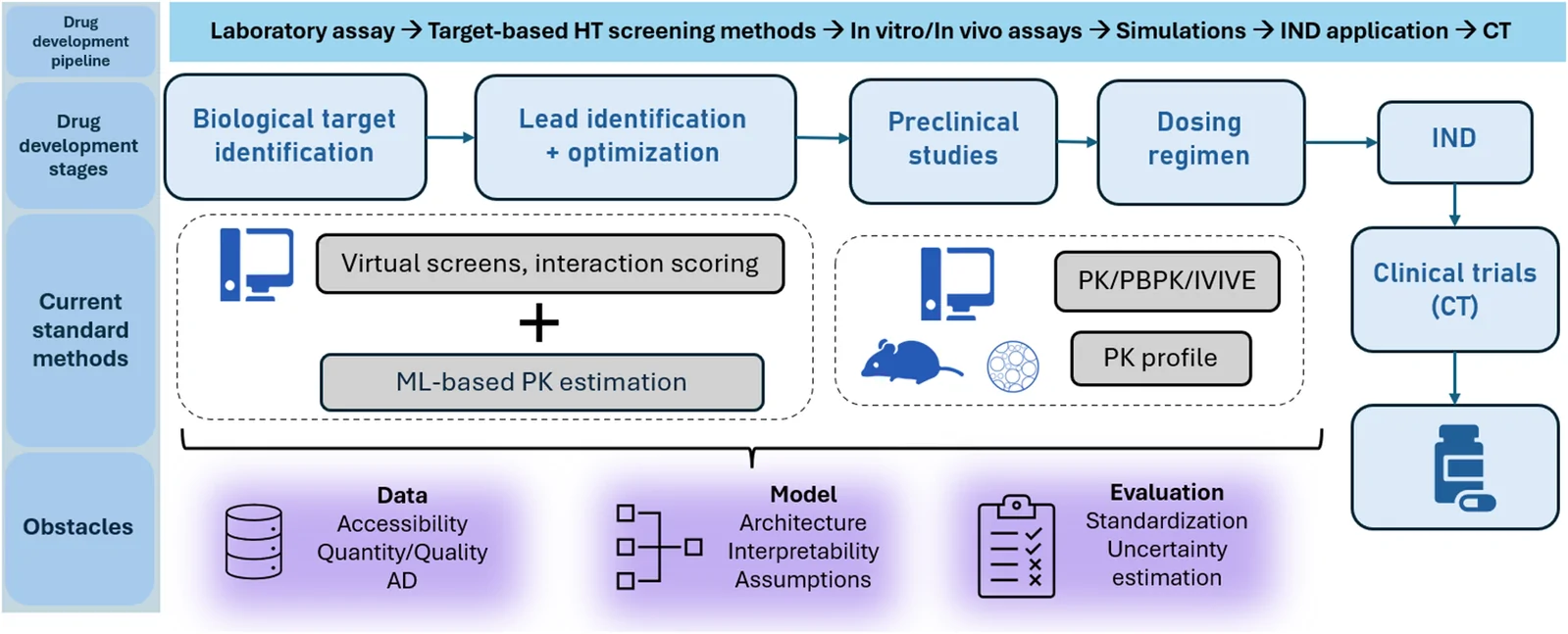
Simplified drug development workflow^2^ with barriers identified. The pipeline begins with target selection and validation, proceeding to compound screening and lead selection through various machine learning (ML) approaches such as high throughput screening. During the lead optimization process, preclinical studies, and dosing regimen design, quantitative pharmacology approaches such as *in vitro-in vivo* extrapolation (IVIVE), pharmacokinetic (PK), and physiologically based pharmacokinetic (PBPK) modeling techniques are used to refine the selected compound with the highest expected efficacy and lowest toxicity in humans prior to submission of an investigational new drug application (IND). Upon approval, human clinical trials (CT) may begin for safety and market potential evaluation. Current standard methods for target identification and lead optimization via computational virtual screening and ML-based PK estimation traditionally proceed to preclinical studies where PBPK simulation and PK profile estimation necessitates in vivo data or in vitro scale-up methods. These techniques and their combined application tend to share common obstacles relating to data, model architecture, and evaluation methods. This review highlights these barriers and offers recommendations to scalable computational PK prediction techniques.

Target-based drug discovery employs quantitative structure–activity relationship (QSAR) methods with the central claim that drugs with similar structure will behave similarly in a biological system. Lead optimization is a critical stage of the drug development process where hits are refined by assessing their structure–activity relationships with the target. Then *in vitro* and *in vivo* properties are measured or extrapolated with the goal of developing a dosing regimen and preparing an investigational new drug (IND) application prior to human CTs.^4^ Drug pharmacokinetic (PK) property estimation during lead optimization is crucial in ensuring the successful and safe deployment of a novel therapeutic in CTs. Poor PK profiles can lead to insufficient drug exposure of target tissues and/or severe complications due to toxicity.^5^ Pharmacokinetic techniques can model drug absorption, distribution, metabolism, and excretion (ADME) through the body and produce exposure curves for summary PK parameter evaluation, like area under the concentration curve (AUC).^6,7^ The traditional approach to determine time-exposure curves in this modeling process is through mechanistic techniques such as physiologically based pharmacokinetic (PBPK) modeling.^8^

Classical PK mechanistic models consist of a central compartment and one or more peripheral compartments that are related via rate constants. In PBPK modeling techniques additional compartments may be present to account for drug distributions into different organs or tissues which are connected by flow rates.^8^ Traditional PBPK models rely on previously measured empirical values as input parameters. Allometric or single species scaling, types of empirical scaling methods, may also be applied to infer human PK parameters from measurements in preclinical species or *in vitro* based on body weight and organ volume relationships.^9^ *In vitro-in vivo* extrapolation (IVIVE) is an additional approach used to model the physiological and biochemical environment with organ blood flow rates, elimination rates and drug partitions.^5^ These mechanistic models have been traditionally used to estimate PK parameters and full PK profiles based on a set of ordinary differential equations (ODEs). PBPK models specifically offer an effective prediction method of concentration–time (*C*–*t*) profiles and associated interpretability of new pharmaceuticals essential for designing an appropriate dosing regimen (Figure 1).^8^ These models aim to establish a mechanistic understanding of compound behavior in an organism, select promising hits, and reduce risk benefit ratios.^10^

PBPK mechanistic models use drug characteristics typically obtained from cell or animal-based studies.^10^ Input parameters into these models may be challenging to derive for novel compounds and may vary or even contradict depending on the method used to obtain these input parameters. Animal scaling methods include species scale-up, the Øie-Tozer equation, or multiple linear regression are popular techniques.^11^ Drug ADME properties that are independent from the physiological processes of the organism such as solubility, molecular weight, acid dissociation constant values, or lipophilicity are inherent physicochemical properties of the chemical,^10^ which can be estimated with computational methods using machine learning (ML) algorithms with various virtual representations of the compounds.^12–15^ Whereas drug PK properties such as unbound drug fraction *f*_u_, tissue-plasma partition coefficient, and bioavailability (*F*) depend on organismal physiology and are more difficult to deduce from molecular properties alone.^10^ These PK parameters are used as drug-specific inputs into PBPK models and are used downstream to determine *in vivo* PK parameters like clearance (CL), volume of distribution at steady state (*V*_dss_), and *f*_u_.^10^ There is limited high quality data for accurate PK simulations especially on investigational compounds due to the cost and time associated with the direct measurement of these properties in cell culture and live animals. Moreover, variations in measured experimental data and the variations in experimental procedures lead to challenges in obtaining consistent and reliable data. The implementation of computational prediction models is therefore highly desirable in their speed, accuracy, and cost efficiency in the drug discovery pipeline. Additionally, the recent announcement by the National Institutes of Health on allocating funding toward the validation of nonanimal models in an effort to reduce animal testing and find alternatives to models that currently do not translate well into humans highlights the need to advance these computational tools.^16^

Coupling target-based drug discovery techniques with methods estimating *C*–*t* data and PK parameters has the potential to rapidly accelerate the drug development process and reduce costs by avoiding the pursuit of leads with unfavorable PK properties. While traditional PBPK and IVIVE approaches require *in vitro* or *in vivo*-derived parameters, studies began using ML-predicted input parameters using SMILES strings for mechanistic models to determine PK profiles.^17^ The studies found great success with highly correlated predicted parameters compared to experimentally measured values.^17,18^ However, the fundamentally different nature of ML and traditional PK has hindered their widespread combined adoption in drug development pipelines.^19^ The hybrid models serve as a start for ML-based PK profile prediction for physiologically dependent parameters early in the drug discovery process.

This scoping review focuses on identifying conventional ML (CML), deep learning (DL), neural network (NN), and combination ML-mechanistic predictive modeling approaches and highlights the barriers current techniques face. While overviews regarding the integration of ML into quantitative pharmacology models have been published, there is a need to parse the current architectures and limitations in detail to improve these models, assess their reliability, and foster discussions for better model development. The integration of ML into mechanistic models or replacing the ODE element with NNs has been shown to be more computationally efficient for at scale compound screening.^20^ Three main topics are addressed during this analysis: (i) current workflows and pipelines of PK property prediction using ML and a combination ML-mechanistic approaches, (ii) shortcomings of current models predicting PK properties and, (iii) suggestions for superior model development. Improved model reliability is expected to drive greater adoption of *in silico* approaches in early drug development, enabling the prioritization of narrower, higher-quality compound sets before preclinical testing thereby enhancing overall efficiency.

## 2 Methods

This scoping review follows the Preferred Reporting Items for Systematic reviews and Meta-Analyses extension for Scoping Reviews (PRISMA-ScR) guidelines.^21^ Literature searches were carried out in the following databases: Ovid Medline, Embase, and Web of Science. Study collection, management and duplicate removal were performed via the EndNote citation management software,^22^ while additional deduplication, title and abstract screening, and article selection was done using the Covidence web service.^23^

Medical subject heading terms were extracted from key papers identified during the preliminary research process (Appendix I). Additional keyword search terms were collected by the review team to ensure a comprehensive literature search. Optimal keywords were then identified from a preliminary search of the key papers in Ovid Medline to select common medical subject headings to include in the search strategy. The following keywords were included for the Ovid Medline search: “pharmacokinetics”, “biological models”, “pharmaceutical preparations”, and “drug discovery”. “Machine learning” and “deep learning” keywords were not applied directly in the Ovid Medline database search as they excluded key papers representing core ML-mechanistic articles (Appendix I) identified during the preliminary search process. Additionally, “artificial intelligence” and related terms introduced significant noise among the search articles outside the scope of this study. The search was limited to abstracts, English language records, and publication year from 2018 to 2025. The Embase search included emtree terms “pharmacokinetic modeling software” and “machine learning” and were also limited to studies from 2018 through the search date (08/29/2025) and limited for records present in Embase and not in Medline. Similarly, the Web of Science search contained the keywords “pharmacokinetic modeling software” and “machine learning”, and publications were limited to the same date range, English language, and articles as the publication type. Mechanistic only studies were not considered and population PK studies were excluded as to preserve alignment with early drug discovery objectives and filter out articles focusing on the quantification of interindividual variability and covariate effects (for complete search strategy of all three databases, refer to Appendix II).

The database searches were performed by the primary reviewer (LT) with assistance for search strategy development from a library science specialist (DS). Following the collection of records in Covidence web service, abstract screening was conducted by two reviewers and disagreements resolved via consensus (LT and VB). Full text screening was performed by the primary reviewer (LT). The inclusion and exclusion criteria applied limitations on publication types, input data types, predicted parameters, and model development methods. This review focuses on small molecule inputs and predicted PK or ADME properties prioritizing ML models over traditional quantitative pharmacology approaches while including combination ML-mechanistic techniques (for complete inclusion/exclusion criteria see Table 1 for full text screen outside scope exclusion criteria application, refer to Appendix IIa). The study screening and selection process is documented in the PRISMA flowchart with reasons for study exclusion (Figure 2). The year selection is based on Dr. Niazi’s thorough review on the state of AI and ML integration into drug discovery.^24^ The bidirectional encoder representations from transformers (BERT), developed in 2018, revolutionized the area of natural language processing which accelerated research in the AI space for drug development. Additionally, studies focusing on the integration of AI methods throughout the coronavirus disease 2019 (COVID-19) pandemic surged dramatically, and this review aims to capture this period as well.^24^ Furthermore, the application of DL methods for QSAR problems has increased significantly in 2018 then further in 2019.^25^ Studies investigating therapeutic agents that do not qualify as small molecules, such as monoclonal antibodies, peptides, proteolysis targeting chimeras (PROTACs) were excluded (Table 1). Extracting relevant information from each study was performed by the primary reviewer (LT) using a data charting form recording information on the publication (title, authors, journal, date of publication), input data source and features, model development, predicted parameter(s), evaluation, limitations, and additional discussion notes.

**Table 1:** Inclusion and Exclusion Criteria Summary for Record Selection^a^

Record elements	Inclusion criteria	Exclusion criteria
Publication	English, 2018 or after	Review articles
		
Molecule types	Small molecules	Nonsmall molecules (e.g.,: mAbs, PROTACs, multi molecule delivery systems, nanoparticles)
		
Predicted parameter	PK properties, PBPK model input parameters, ADME properties	PK/ADME properties predicted by previously established tools, toxicity prediction only, drug–drug interaction prediction
		
Model	Predictive models, combination/hybrid models, ensemble models	Mechanistic only model
		
		
*Models outside of scope*	Population PK and covariate selection models, single target studies, single drug studies, drug–drug interaction studies	

a This review focuses on small molecule as input into ML or combination ML-mechanistic models predicting PK or ADME parameters. Abbreviations: pharmacokinetic (PK), physiologically-based pharmacokinetics (PBPK), absorption/distribution/metabolism/excretion (ADME), monoclonal antibodies (mAbs), proteolysis targeting chimeras (PROTACs), machine learning (ML).

**Figure 2:**
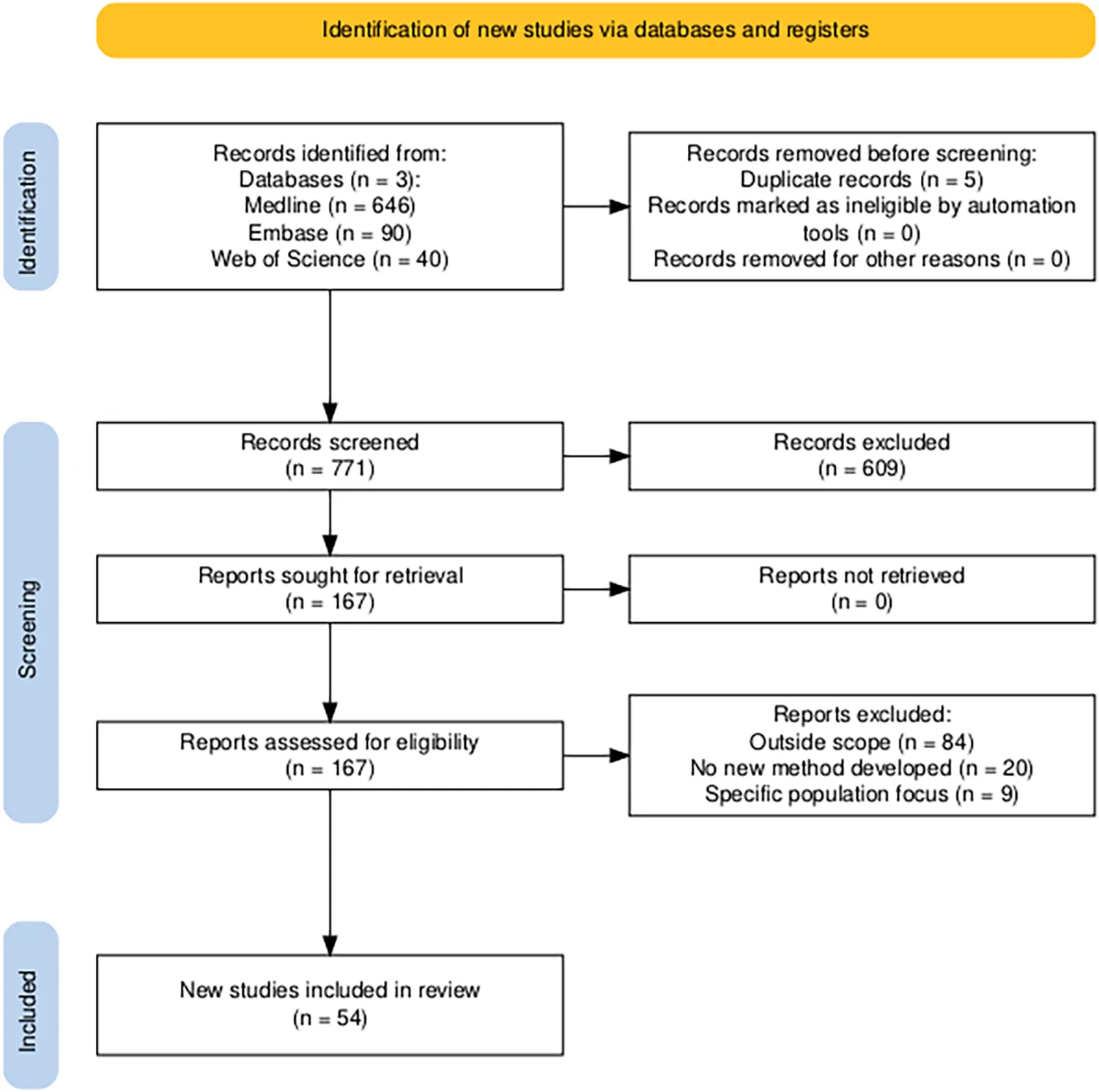
PRISMA flow diagram for record selection. From initial collection of 776 records, five records were removed during deduplication, and 717 articles were excluded by reviewers, resulting in 54 articles selected for analysis. Created using PRISMA2020.^26^

## 3 Results

### 3.1 Article Selection

We retrieved 776 records from the three databases (Medline *n* = 646, Embase *n* = 90, Web of Science *n* = 40). EndNote citation manager identified four duplicate records, and Covidence detected and removed one additional duplicate upon import. Title and abstract screening deemed 609 articles irrelevant leaving 167 records that were sought for full text retrieval. Delivery+, the University at Buffalo library’s service was used to obtain full text articles that were not open access (*n* = 14). The full text screening process removed articles in which there were no new computational methods developed (*n* = 20), studies focused on specific population dosing (*n* = 9), and studies which were outside of the scope of this review (*n* = 84) which included drug–drug interactions studies, single drug/target studies including single drug classes or compounds with a shared mechanism of action, studies using mechanistic models only, studies where no systemic PK property is being predicted, studies using published tools to predict PK properties for further analysis, toxicity end point only studies, and population PK models focusing on covariate selection. The final review includes 54 records. See Figure 2 for a visual depiction of the record selection process.

### 3.2 Prediction Model Architecture

The primary objective of this review is to synthesize current trends, methodological patterns, and knowledge gaps in ML and ML-mechanistic approaches for PK property prediction. Given the substantial heterogeneity across studies in data sets, end points, curation procedures, descriptors, and validation strategies, direct quantitative comparison of reported model performance is often not feasible. Individual studies are summarized to provide methodological context, while section-specific synthesis and concluding remarks focus on broader insights regarding model architectures and data characteristics. ML only methods were employed by close to two-thirds (65%, 35/54) of the selected records without mechanistic models except for performance comparison purposes. Nineteen studies (35%, 19/54) harnessed the power of combination ML-mechanistic models for prediction of PK parameters, summary PK properties, or PK profiles.

#### 3.2.1 Conventional Machine Learning Models

CML methods remain widely used for predicting PK/ADME properties of small molecules. Here, we provide a brief overview of the most commonly applied CML techniques presented among the selected articles (Figure 4). Ensemble decision tree (DT) methods are particularly prominent. Random forests (RF) and their variants (cluster RF, repeated RF aggregate predictions from several perturbed DTs trained on bootstrapped subsets of data. This method leads to robust performance and reduces overfitting.^27^ Gradient boosting (GB) approaches and variants, extreme gradient boosting machine (XGBoost), light gradient boosting machine (LightGBM), and categorical boosting machine (CatBoost), build DTs sequentially, correcting earlier errors. GB consistently delivers high performance in QSAR modeling and broader predictive tasks. Support vector machines (SVMs) function by identifying optimal hyperplanes separating data points and scale well to large data sets with high dimensional descriptor spaces.^28^ Gaussian processes (GP) offer strong theoretical support, provide uncertainty estimates, though their computational cost can limit their application to small data sets.^29^ Partial least-squares (PLS) models can be particularly effective at selecting the most meaningful parameters from a set of features, and are efficient at handling multicollinearity.^30^ The *k*-nearest neighbors algorithm (kNN) provides a simple nonlinear ML method, making predictions based on similarity to nearby compounds in the descriptor space^31^ (Figure 4).

Across the 35 ML focused studies, 12 used CML approaches serving as standalone or ensemble predictive tools. Others used CML models as baselines for evaluating DL and hybrid frameworks (Figure 3, Table 2). In the following sections, the primary approach to prediction is described and relevant findings are summarized from selected articles.

**Figure 3:**
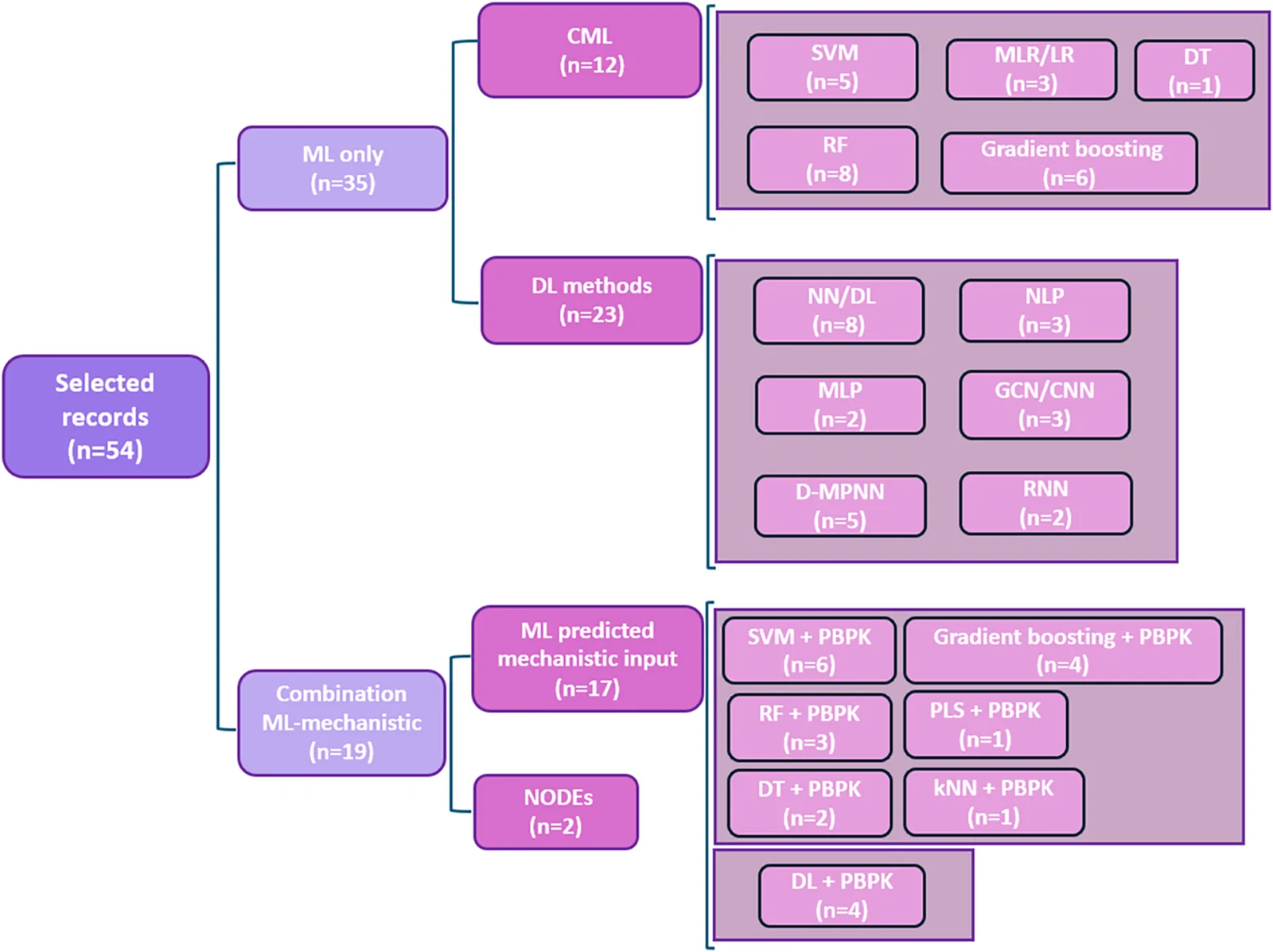
Breakdown of model architectures of selected records. Abbreviations: machine learning (ML), conventional ML (CML), *k*-nearest neighbor (kNN), support vector machine (SVM), multiple linear regression/linear regression (MLR/LR), decision tree (DT), random forest (RF), partial least-squares (PLS), deep learning (DL), neural ordinary differential equations (NODEs), neural network (NN), multilayer perceptron (MLP), graph neural network (GNN), convolutional neural network (CNN), deep message passing neural network (D-MPNN), recurrent neural network (RNN), natural language processing (NLP), physiologically based pharmacokinetic model (PBPK).

**Table 2:** Distribution of Selected Records among Main Model Mechanisms Investigated^a^

Main model mechanism	Model name	Records	Output
CML-ADME	–	^32^	BBB permeability
	CypReact	^33^	CYP enzyme substrate
	MDCKpred	^34^	Apparent permeability
CML-PK	–	^41^	Human F
	–	^35^	Fraction unbound drug in brain homogenate
	–	^36^	Unbound brain-to-plasma concentration ratio
	–	^37^	Rat total plasma CL, microsomal unbound fraction
	–	^38^	Human *V*_dss_
	cpVss	^11^	Human *V*_dss_
	–	^39^	Human *F* (po), CL (iv), *V*_dss_ (iv), AUC (iv), AUC (po), *C*_max_ (po), *t*_1/2_ (iv), *t*_1/2_ (po), *t*_max_ (po), *V*_dss_/*F* (po), CLR (iv), CLR (po)
	–	^40^	Rat CL, *V*_dss_, *C*–*t* profile
	Global QSAR	^42^	Rat *V*_dss_, tissue-to-plasma partition coefficient
NN/DL	Deep-Tensor	^45^	Human total CL
	Multimodal Deep-Tensor	^46^	Human total CL, *V*_dss_
	–	^47^	Human plasma protein binding
	–	^49^	Human fraction unbound in plasma
	–	^50^	Human renal excretion of drug unchanged in urine, renal CL
	–	^52^	Human/rat/mouse microsomal CL
MLP	NLSD models	^54^	CYP enzyme substrate
	–	^55^	Human effective permeability
GCN/CNN	ML+DeepSnap-DL	^56,57^	Rat CL
	–	^58^	CYP enzyme substrate
D-MPNN	MONSTROUS	^59^	Human transporter inhibitor/substrate
	–	^60^	Rat F (po), CL (iv), *V*_dss_ (iv), AUC (iv), AUC (po), *C*_max_ (iv), *C*_max_ (po), *t*_1/2_ (iv), *t*_1/2_ (po)
	–	^61^	Mouse/rat/human liver microsomal assay, plasma protein binding, hepatocyte stability, mouse/rat CL, *V*_dss_, *F*, Log *D*, solubility, Caco2 cell permeability and efflux ratio
	–	^85^	Mouse tissue-to-plasma partition coefficient
	PredPS	^65^	Compound stability in human plasma
	PKStack	^9^	Human/rat/mouse/dog/monkey *t*_max_, *C*_max_, AUC (iv), AUC (po), *t*_1/2_ (iv), *t*_1/2_ (po), CL, *V*_dss_, *F*
RNN	RNN-BBB	^25^	BBB permeability
	DeepNCA	^67^	Human *t*_1/2_, AUC, *C*_max_
NLP	–	^69^	Human CL, *V*_dss_
	–	^68^	Human (po) AUC, *C*_max_, *t*_max_, *t*_1/2_, CL/F, apparent *V*_dss_ from central compartment, MRT, F
	–	^72^	BBB permeability
Combined ML + mechanistic	–	^73^	Rat plasma/hepatic/renal AUC (po), *C*_max_ (po)
	–	^18^	Human *C*_max_, AUC
	–	^74^	Human intrinsic/plasma CL
	–	^75^	BBB permeability
	–	^84^	Rat ratio unbound drug concentration in brain ECF over plasma at steady-state
	–	^76^	Rat AUC (po), *C*_max_ (po), AUC (iv), *t*_1/2_, CL, *V*_dss_, MRT, *F*, AUMC
	–	^86^	Mouse *V*_dss_, *F*, first-order rate constants (ka, k12, k21, k10)
	–	^78^	Human *F*
	ANDROMEDA	^77,79,88^	Human fraction absorbed from the gastrointestinal tract, *F*, *t*_1/2_, dissolution potential, fraction unbound, *V*_dss_, CL (po), biliary/hepatic/renal/intrinsic CL, fraction excreted renally
	mTPA	^80^	End points driving efficacy
	–	^81^	End points driving efficacy
	–	^82^	Rat F, fraction absorbed
	CLglb	^83^	Rat intrinsic CL
	–	^20^	Rat F, AUC (po), AUC (iv)
	–	^85^	Mouse first-order rate constants (*k*_in_, *k*_out_, *k*_deep,br,in_, *k*_deep,br,out_)
NODEs	–	^19,87^	*C*–*t* profiles

a Abbreviations: machine learning (ML), conventional ML (CML), absorption/distribution/metabolism/excretion (ADME), pharmacokinetics (PK), deep learning (DL), neural ordinary differential equations (NODEs), neural network (NN), multilayer perceptron (MLP), graph neural network (GNN), convolutional neural network (CNN), deep message passing neural network (D-MPNN), recurrent neural network (RNN), natural language processing (NLP). Additional abbreviations: blood brain barrier (BBB), cytochrome p450 (CYP), bioavailability (*F*), clearance (CL), volume of distribution at steady state (*V*_dss_), oral administration (po), intravenous administration (iv), area under the curve (AUC), maximum concentration (*C*_max_), half-life (t1/2), concentration time (*C*–*t*), renal clearance (CLR), time at maximum concentration (*t*_max_), mean residence time (MRT), extra cellular fluid (ECF), area under the moment curve (AUMC).

Three studies applied CML (Figure 3, Table 2) models to predict ADME or physicochemical properties of small molecules. Yuan et al. developed SVM-based blood brain barrier (BBB) permeability classifiers using two data sets, multiple descriptor and fingerprint types, and various data splitting strategies. The authors combined property-based descriptors calculated from three-dimensional structures and fragment-based one and two–dimensional descriptors. The octanol–water distribution coefficient at pH 7.4 (log *D*) correlation was used as baseline, the Kennard-stone splitting yielded the best generalization, and the original nonextended data set gave higher correlations with fragment-based descriptors consistently outperforming alternatives.^32^ The Kennard-stone splitting selects the center of the chemical space using Euclidean space based on descriptor-derived properties. Then the furthest compound is selected with this process continuing until reaching the selection needed for training. This method is hypothesized to cover the chemical property space uniformly and produce a representative subset for training.^32^ Tian et al. compared their ensemble model, CypReact, composed of SVM, linear regression, DT, and RF components, with published cytochrome P450 enzyme (CYP) reactivity predictors. CypReact outperformed baseline models and SMARTCyp-React for three out of nine enzymes and surpassed ADMET Predictor for all CYP models.^33^ Finally, Patel and colleagues developed a unique molecular dynamics-informed classifier for Madin-Darby Canine Kidney (MDCK) cell permeability (*P*_app_), a surrogate for human intestinal absorption. They simulated a dimyristoylphosphatidylcholine (DMPC) membrane to obtain its minimized structure, computed interaction-based descriptors between small molecules and the membrane fragments and used these features to classify compounds by *P*_app_.^34^ Yuan^32^ and Patel^34^ focused on descriptor space optimization while Tian^33^ emphasized a novel model learning mechanism to break through performance ceilings. Such descriptor and algorithm engineering measures are complementary and move the field toward drug discovery application scenarios, especially with highly interpretable descriptors in medicinal chemistry workflows such as the ones developed for MDCKPred.^34^

A further nine articles used CML (Figure 3, Table 2) methods to predict compartment model input parameters or PK properties. Esaki et al. evaluated RF, GB, SVM, and PLS models for predicting fraction unbound drug in homogenized brain tissue (*F*_u,brain_). GB achieved the lowest root mean squared error (RMSE) and percent fold error followed by SVM and RF.^35^ Similarly, Umemori et al. modeled the unbound brain-to-plasma concentration ratio (*K*_p,uu,brain_) with RF trained with combinations of descriptors and commercial software-predicted brain penetration related parameters (PBrPs). Incorporating the full BPrPs set substantially reduced the RMSE suggesting that *in silico* predicted parameters can meaningfully enhance predictive accuracy of the model.^36^ Kosugi et al. developed PLS, SVM, GP, and RF models predicting total clearance (CL_tot_) and microsomal unbound fraction (*f*_u,mic_) in rats. The RF and SVM models with radial basis function were superior at *CL*_*tot*_ prediction, and GP with hyperparameters from forward variable selection and rescaled procedures performed best for predicting *f*_u,mic_.^37^

Summary PK parameters and *C*–*t* curve prediction are traditionally performed by mechanistic models, however, studies explored the possibility of using CML techniques only to avoid mechanistic assumptions made by compartment models. Lombardo et al. found that a RF parametrized with a few select descriptors outperformed the model trained with more descriptors in predicting *V*_dss_, emphasizing the need for selecting essential features and reducing noise.^38^ The Spjuth group also predicted *V*_dss_ by employing conformal prediction (CP), an augmented ML technique with confidence prediction and an underlying SVM architecture. CPVss showed comparable performance to animal-based models and outperformed the Rodgers-Lukocova PBPK method. Uncertainty estimation is not assessed by most models whereas CPVss allows for user-defined confidence interval adjustment and outputs prediction intervals over point estimates^11^ addressing a critical gap in the field. Miljkovic et al. predicted additional parameters such as *F*, volume of distribution after oral administration (*V*_dss_/*F*), time at maximum concentration (*t*_max_), CL, and *V*_dss_ after i.v. administration, and AUC, *C*_max_, half-life (*t*_1/2_), and renal clearance (CLR) for both p.o. and i.v. administration. The analysis also showed RF having a slight advantage over XGBoost.^39^ Pillai et al. predicted CL, *V*_dss_, and *C*–*t* curves in rats using RF, SVM, and GB. XGBoost was the top performing model that best captured both mono- and biexponential PK relationships.^40^ Yang et al. applied RF, XGBoost, CatBoost, and LightGBM to predict oral bioavailability (*F*), a complex yet critical parameter for PK clinical efficacy. The RF model yielded the lowest errors, and highest correlation with observed values on the external validation.^41^ Finally, Mathew et al. completed an evaluation of their rat global QSAR model using GB against three PBPK models (Poulin & Theil, Rodgers & Rowland, Lukacova), a tissue-to-plasma coefficient (*K*_p_) scalar method, *in vitro* methods for calculating *V*_dss_ and *K*_p_, equivalency methods, Øie-Tozer methods, and scaled PBPK methods in predicting rat *V*_dss_ and *K*_p_. The global QSAR model performed best in *V*_dss_ prediction followed by Øie-Tozer from dog or nonhuman primate data.^42^

Ensemble methods dominate the field of CML-based ADME/PK prediction, particularly via tree-based ensemble methods like RF and XGBoost that were consistently the top performing models in our analysis. Kosugi and colleagues presented the only exception to this pattern with their radial basis function CL prediction model.^37^ Four articles directly compared QSAR-based approaches to conventional mechanistic PBPK and scale-up methods and noted that the ML-based approaches performed best. The authors listed the sensitivity to input parameters and physical assumptions made by mechanistic models being the main culprits.^11,37,40,42^ Interestingly, Miljkovic et al. explicitly tested DL methods against tree-based ensemble techniques and determined that for highly variable *in vivo* PK data and limited data availability applications DL techniques offered no advantage.^39^ Data augmentation and feature set engineering has also taken center stage in this section. Umemori et al. demonstrated an approach to overcome the sparsity of PK data sets by software predicted parameter supplementation showing significant accuracy improvement evaluated by RMSE.^36^ On the other hand, Lombardo and colleagues demonstrated that in some cases sparser descriptor sets may provide comparable performance while maintaining a higher degree of interpretability,^43^ though combination descriptors such as fingerprints coupled with physicochemical descriptors showed clear performance benefits by others.^39^

#### 3.2.2 Deep Learning Architectures

DL methods such as NNs, inspired by neurological pathways, can learn complex patterns directly from chemical structures (Figure 4). In this review, neural networks were the most frequently utilized ML models (66%, 23/35; Figure 3, Table 2), consistent with prior reportings of NNs being the predominant ML approach in QSAR problems.^44^ These architectures effectively handle complex nonlinear relationships of large dimensional features present in molecular data.^25^ Iwata et al. built their multimodal Tensor model using deep neural networks (DNNs) to predict CL_tot_ and *V*_dss_, performing better than baseline mechanistic scale up methods. The authors integrated chemical structure and nonclinical data into a multimodal prediction framework,^45^ then used the strategy to impute missing data, establishing a powerful technique for handling incomplete data sets.^46^ Han et al. compared consensus and ensemble NNs to predict plasma protein binding (PPB). The associative neural network, an ensemble architecture with a single hidden layer NN and kNNs, outperformed other ML techniques.^47^ Czub et al. employed an ensemble model approach by integrating three Catboost models, and two NNs into a regression model as well as three CatBoost models, two NNs, one extra tree, and one RF into a classification model predicting human intestinal absorption. The regression model performed well, however, the classification model presented with a high false positive rate on the test set suggesting lack of generalizability to unseen data.^48^ Watanabe et al. evaluated fraction of drug unbound in plasma (*f*_u,p_) prediction using RF, SVM, kNN, NN, Adaboost, and PLS in a three class classification task where RF outperformed all other models,^49^ demonstrating the powerful utility of RFs even against NNs. Among their regression models evaluated on the linear scale SVM showed the lowest error rate while RF performed the best overall on the log scale.^49^ Watanabe further developed an approach to predict human renal excretion of different compound types using SVM, NN, PLS, and RF. The RF models continued to show superior performance for renal excretion and for intermediate and secretion compound types, while the PLS architecture performed best for reabsorption types.^50^

**Figure 4:**
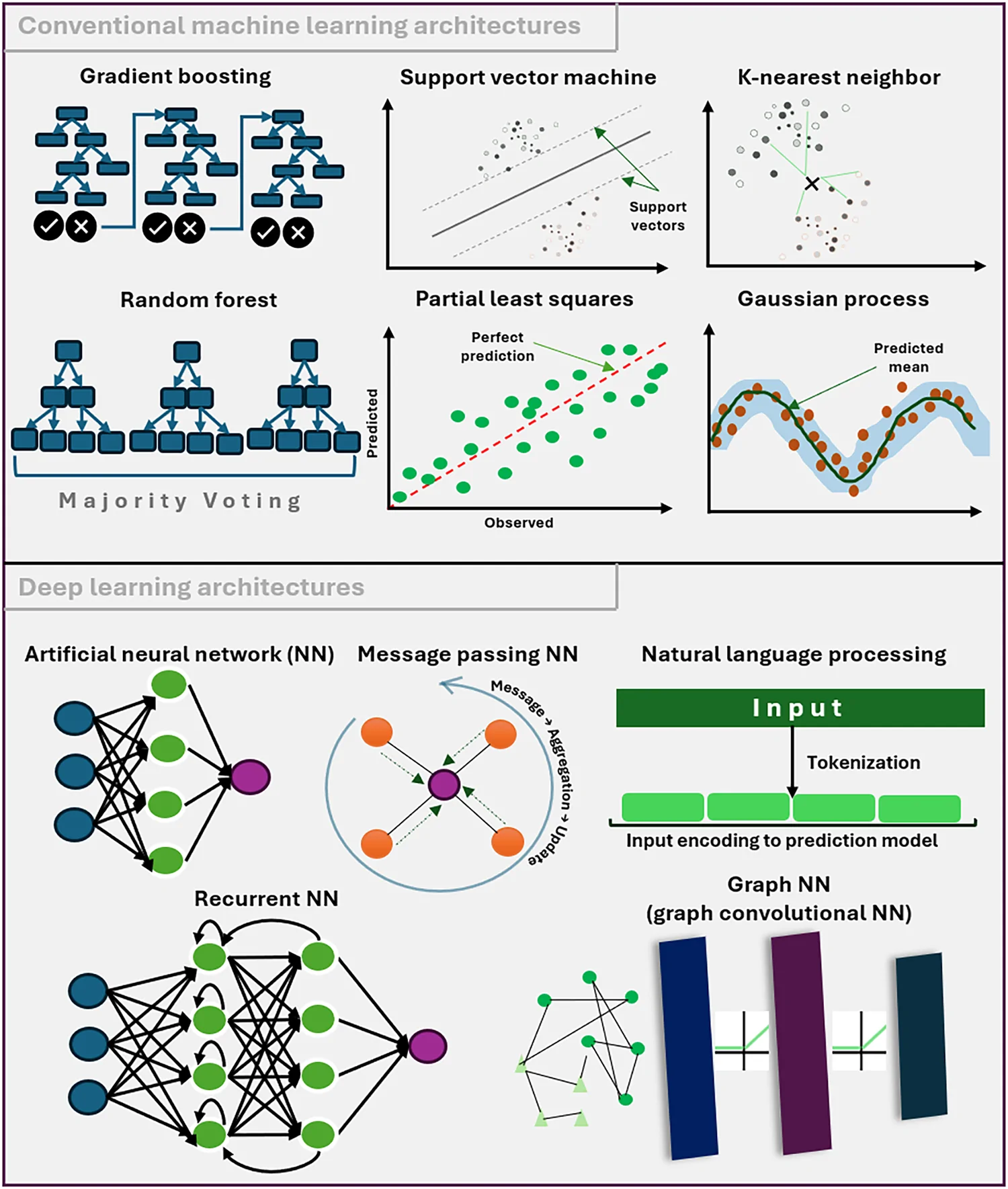
Overview of conventional machine learning (CML) (top) and deep learning (DL) (bottom) architectures used in selected studies. CML methods include gradient boosting decision tree (DTs) methods (e.g.,: XGBoost, LightGBM, CatBoost), which iteratively build ensembles of weak learners to minimize prediction error; support vector machines (SVMs), which identify optimal hyperplanes separating data in high-dimensional space; *k*-nearest neighbor (kNN), which assign predictions based on similarity to local data points; random forests (RFs), which aggregate predictions from multiple decorrelated DTs; partial least-squares (PLS), which project predictors into latent variables to maximize covariance with response; and Gaussian processes (GP), which model data via probabilistic distributions over functions. DL architectures include artificial neural networks (NNs) such as forward pass multilayer perceptrons (MLPs); recurrent NNs (RNNs) which retain information through sequential hidden states; graph NNs (GNNs), which operate on graph structured data using approaches such as graph convolutions (GCNNs) and message passing (MPNN) to aggregate information from neighboring nodes; and natural language processing (NLP) models, which process tokenized input sequences. Various techniques can be combined into ensemble or consensus models leveraging application-specific advantages to overcome inherent assumptions from individual architectures and improve performance and/or generalizability for machine learning-based pharmacokinetic (PK) property prediction.

Multitask (MT) models predict output parameters for more than one end point as opposed to single-task (ST) models predicting a single output variable. MT has been regularly applied in QSAR modeling in the past decade. This method can leverage information transfers across tasks via joint representations typically outperforming ST counterparts.^51^ Wenzel et al. leveraged MT learning and ST DNN models predicting human, rat, and mouse microsomal clearance where the MT approach was the top performer in most tasks among the three species. However, Caco-2 cell permeability prediction was not improved by the MT approach.^52^

Multilayer perceptrons (MLP) are fully connected NNs, describing fundamental architectures where hidden layer neurons are connected to all neurons in the subsequent layer and information passes through in the forward direction providing further flexibility for modeling nonlinear features. MLPs are a subclass of DL models, especially when containing multiple hidden layers between input and output layers (Figure 4). Case studies modeling nonlinear QSAR relationships showed that MLPs achieved better performance than PLS, quadratic PLS, and multiple linear regression models.^53^ Two records implemented MLP using feedforward NNs directly driving their prediction architecture. Shan et al. compared kNN baseline architecture against, five network-based label space division (NLSD) models (MLP, XGBoost, extra tree, RF, SVM) to predict CYP enzyme substrate selectivity but found that the NLSD-XGBoost model outperformed all others in both cross validation and hold out tests. In fact, the NLSD-MLP approach did not perform better than any other NLSD algorithms in this multilabel CYP selectivity prediction.^54^ Nevertheless, Gatarić et al. integrated MLP for predicting *in vivo* human intestinal permeability values (*P*_eff_). Predictions achieved a cross validation coefficient of 0.8 and correlated well with reported fraction absorbed values, however, no baseline model was used for evaluation.^55^ While the utility of MLP has been applied in QSAR contexts,^53^ PK/ADME property prediction has not been sufficiently evaluated.

Convolutional neural networks (CNNs) traditionally offer advantages in image processing tasks. Molecules can also be processed as two-dimensional images using this technique with exceptional performance^27^ (Figure 4). Harnessing this strategy, Mamada et al. constructed their DeepSnap-DL model using images of molecules as input features rotated at various angles into a CNN to capture three-dimensional structural information to predict rat clearance. The study design included combination DeepSnap-CML models in the form of an ensemble model, averaging prediction values of the DL and ML models, and a consensus model, taking the agreement between DL and ML. The consensus model performed best, followed by the ensemble models, then DeepSnap with molecular descriptors only.^56,57^ While the models trained on the CNN-based descriptors outperformed standard two-dimensional molecular representations, the high-performing consensus model was unable to evaluate all compounds with the authors noting this as a limitation in early screening scenarios.^56^ Wu et al. also applied CNNs and compared RF, GB, and DNN in CYP enzyme selectivity prediction. XGBoost performed best on CYP450 enzymes 1A2, 2C9, 2C19, and 2D6 while DNN accuracy was superior on the 3A4 enzyme.^58^

Chemprop, an open source chemical property prediction framework, uses a deep message passing neural network (D-MPNN) architecture where atoms are represented as nodes and bonds between them as edges (Figure 4). Chemprop follows two main steps: the message passing phase refining atomic and bond features, and the readout phase, creating the learned representation of a molecule.^59^ Abdulhameed et al. built MONSTROUS, a graph convolutional neural network (GCNN or GCN) using Chemprop to predict transporter inhibitor or substrate status of drugs. GCNNs harness the contextual strength of graph representations of molecules by combining graph neural networks (GNNs) and the convolution operations of CNNs to learn molecular structure more accurately.^59^ Obrezanova et al. also exploited the power of Chemprop to predict rat F, CL, *V*_dss_, and AUC, *C*_max_, and *t*_1/2_ both after i.v. and p.o. administration. They compared the D-MPNN model to a GB, GP, CP, and MT and ST NN methods. The Chemprop and NN methods performed best for most end points except for p.o. *C*_max_ where GP dominated.^60^ Similarly, Walter et al. aimed to predict mouse, rat, and human PK end points using ST and MT Chemprop models against baseline RF. MT Chemprop outperformed stacked RF, RF, and ST Chemprop except for log *D* assay prediction, where there was no significant performance difference between ST and MT Chemprop.^61^ Handa et al. also used RF, MT Chemprop, as well as the repeated RF architecture to predict tissue-to-plasma partition coefficient (*K*_p_), where RF performed best, except for *K*_p_ prediction in liver and adipose tissue where MT Chemprop was the top performer.^62^ CNNs, GNNs, and GCNNs are efficient at encoding node representations, however, neighborhood information is not considered, ignoring long-range dependencies.^63^ Attention mechanisms may overcome these shortcomings.

Attention mechanisms can handle inputs of variable sizes and focus on the most important aspects of the input data.^63^ Graph attention networks (GATs) are a convolution style NN that operate on graph data while leveraging attention layers.^63^ For instance, AttentiveFP is a GNN with an applied graph attention mechanism.^64^ Jang et al. employed an attention-based GNN, PredPS, to predict compound stability in human plasma. They compared their attention mechanism with RF, SVM, D-MPNN, and complex multiplex passing network architectures. The attention-based GNN outperformed conventional ML models (RF, SVM), but the D-MPNN and complex multiplex passing networks were superior in classification accuracy.^65^ Zhang et al. developed PKStack with six ST models (three GB, kNN, SVM, RF), five MT GNNs (GAT, GCNN, MPNN, AttentiveFP, D-MPNN), and an ensemble approach for 45 task predictions (nine PK parameters in five species). The predictions were also fed into a Bayesian NN outputting a probability distribution. PKStack achieved the highest accuracy determined by coefficient of determination (*R* ^2^) and RMSE on 91% of the tasks.^9^

Standard NNs rely on fixed length vectors as inputs assuming no time dependency between data points, therefore the state of the network is lost after processing each sample. Recurrent neural networks (RNN) are feedforward architectures that can utilize information across time steps allowing the model to consider temporal dependencies^44^ (Figure 4). Alsenaan et al. employed an RNN to classify the ability of drugs to pass through the BBB. The RNN architecture showed high accuracy, outperforming other CML and DL methods for BBB permeability from the literature.^25^ Convolutional RNNs combine convolutional spatial feature extraction with sequential modeling, addressing RNN limitations on high-dimensional data while incorporating temporal dependencies often missed by CNNs.^66^ Liu et al. used a convolutional RNN architecture to predict *C*–*t* profiles with their DeepNCA model and compared it to traditional noncompartmental analysis (NCA). Traditional NCA had excellent performance while DeepNCA performed adequately on dense sampling and handled increasingly sparse sampling better than conventional techniques.^67^

Natural language processing (NLP) techniques for molecular representations typically consider compound substructures as words and whole compound structures as sentences for generating a tokenized input^54^ Figure 4. NLP inspired models have been incorporated into PK prediction models (Figure 3, Table 2),^9,68,69^ ChemBERTa being the common choice.^70^ ChemBERTa incorporates the transformer architecture into NLP tasks and has shown to substantially improve model performance.^71^ Kwon et al. tokenized SMILES strings for input sequence generation into ChemBERTa that then produced embeddings for ML model training. This technique improved the model’s performance in predicting human clearance via XGBoost.^69^ Li et al. used ChemBERTa-77M-MTR, an MT variation of ChemBERTa, to predict molecular properties based on SMILES sequences by generating token embeddings for their GNN model, MMPK, predicting human oral PK parameters. The study also employed breaking of retrosynthetically interesting chemical substructures (BRICS) as another molecular representation input modality. These local substructures were then encoded using a graph network integrated into MMPK via a substructure aware cross attention mechanism. Based on geometric mean fold error MMPK outperformed XGBoost, GNN, GCNN, graph isomorphism network, transformer, and GAT. MMPK achieved high accuracy for *T*_max_, *t*_1/2_, mean residence time (MRT), and F, while AUC, *C*_max_, oral clearance (CL/*F*), and drug distribution after oral administration (V2/*F*) prediction was more challenging for the model.^68^ Another study harnessed concepts used by NLP methods to create clique descriptors. The method by Jia et al. breaks molecules down into their substructures and predicts the probability of BBB permeability. The clique descriptor trained RF models with or without synthetic minority oversampling technique (SMOTE) outperformed published NN algorithms, however, showed lower sensitivity scores. Clique trained models with SMOTE also outperformed published models in specificity and Matthew’s Correlation Coefficient. The SMOTE sampling technique aids in the generation of testing and training data sets from imbalanced data sets where the acquisition of true negative samples is challenging. This is particularly prevalent in BBB permeability data sets like B3DB where the frequency of BBB positive samples outweighs negative samples.^72^

Studies find little to no advantage to the deep NN architectures over simpler ML methods in most cases.^39,68^ However, the development of more sophisticated DL strategies were suggested to potentially enhance performance in these tasks^68^ while others pointed to small improvements in optimization with specific architectural designs such as pyramidal style neuron structures.^52^ Small PK data sets ranging in the hundreds to low thousands of compounds allow RF and XGBoost to match or outperform carefully fine-tuned DL models. The strength of DL models shines on large, diverse data, combined architectures, and learned representations. Application of RF and NNs together show clear benefits over using either architecture alone,^48^ and MPNN algorithms such as Chemprop and Alchemite together with XGBoost also performed above DL-only methods.^58^

MT models predicting multiple end points also demonstrate benefits over their ST counterparts^9,52,68^ but these findings have not been consistent across all end points. Walter et al. found training on an industrial set of Log *D* assays, MT showed no benefit over ST architectures,^61^ which may be due to the large *in vitro* data set not offering further information to the MT model over the ST counterpart. However, MT graph representation learning overall show better performance over traditional descriptors.^61^ These graph-based architecture like GNNs, CNN, and D-MPNNs appear to be a continuing highlight in recent articles on PK prediction tasks. The incorporation of attention mechanism has the potential to propel these architectures even further ahead of CML techniques.^9,65^ Multimodal^17,45,46,56,57,59^ and combination DL models with mechanistic components while not incorporating full PBPK models are clearly on the rise as well.^67,72^ Beyond architectural alterations, data quantity and quality are deemed decisive of model performance in most cases. Similar to CML techniques, there is an increased emphasis on data imputation methods to increase training data availability as well as using stacked architectures for missing data imputation, suggesting that data diversity and scale often matter more than model design.^37,62,68,72^ There is a clear shift in the application of DL frameworks toward effective learned representations and moving in the direction of complex MT and multimodal models to battle data scarcity especially for the prediction of closely related end points.

#### 3.2.3 Combination ML-Mechanistic Models

Merging mechanistic approaches and parameter estimation tools using ML can allow for preclinical animal free PK evaluation of putative drug candidates.^73^ ML-mechanistic combination models represented 35% of the selected records (19/54) in this review (Figure 3, Table 2). Among these studies there were two distinct approaches, using ML models to predict input parameters for mechanistic models (89%, 17/19) and neural ordinary differential equations (NODEs) (11%, 2/19). In the following section we summarize the model architectures and main findings of the studies.

Kamiya et al. built a LightGBM model to predict input parameters for a compartment model using chemical descriptors and compared the pipeline to traditional *in vivo*-derived input parameter generation and PBPK model estimation. In their study, they focused on food and industrial chemicals to predict exposure in rats as *in vivo* data may not be readily available for these compounds. *C*_max_ and AUC estimates from *in vivo* and *in silico*-derived parameters correlated with coefficients ranging from 0.83 to 0.87 in plasma, liver, and kidney compartments.^73^ Similar performance was obtained when applied to human plasma concentration values suggesting ML-based input parameter prediction to be a valuable alternative when animal-based data is not available.^18^ In a similar approach, Keefer et al. used XGBoost trained on *in vitro* or *in silico* values coupled with IVIVE models to predict human intrinsic clearance (*C*L_int_) and in vivo plasma clearance (CL_p_). They found the combination ML-IVIVE model using *in vitro* data performed best based on average absolute fold error with the parallel tube liver model outperforming the commonly applied well stirred liver model for IVIVE. Notably, both ML-IVIVE approaches outperformed their mechanistic only counterparts.^74^ Jia et al. trained 15 QSAR models with three ML architectures and five descriptor types. They found that SVM with merged descriptors worked best, which was then applied to a hybrid ML-PBPK model. In contrast to the previous studies,^18,73,74^ the best combination model did not perform better than traditional PBPK modeling with the authors citing error propagation being the contributing factor particularly in cL prediction tasks.^75^ However, their hierarchical ML with QSAR-predicted outputs used as inputs for a second step ML process achieved superior performance to both PBPK and hybrid approaches. The hierarchical ML model being entirely data-driven captures additional patterns of mechanisms of complex biology where models with mechanistic components typically fail to generalize without explicit model adjustment.^75^

Beckers et al. used a DNN to predict compartment model constants for a three compartment PBPK model. Rat *C*–*t* profiles were then predicted followed by NCA to calculate AUC p.o., *C*_max_ p.o., AUC i.v., CL, *t*_1/2_, *V*_dss_, MRT, *F*, and area under the first moment curve (AUMC). Their model, Deep Ct, outperformed others on seven of nine parameters (*t*_1/2_ and MRT better predicted with direct ML prediction). Consistent with previous studies, they found that i.v. end points are predicted more accurately than oral administration parameters.^76^ Fagerholm and colleagues investigated oral PK parameter estimation as well, publishing a series of studies evaluating their CP model (see section Machine Learning Models) in conjunction with a PBPK model for macrocyclic compounds.^77^ Furthermore, they created and tested their ANDROMEDA framework consisting of nine CML models and three structural alert models integrated into two mechanistic PBPK models to predict oral bioavailability.^78^ Overall, incorporating the CP model exhibited an advantage over traditional ML models in offering an effective method for quantifying prediction confidence.^79^

Using a different approach, Chen et al. used PBPK model Monte Carlo simulated *C*–*t* curves to use for ML training to establish a relationship between parameters of the profiles and the responses they elicit. Then a regression DT was applied to create ranges of PK end points that produce various responses. Their approach of model-based target pharmacology assessment demonstrates a proof of concept study that reversing modeling approaches can help decipher pharmacodynamic responses relevant for drug development.^80,81^ Daga et al. also performed PBPK simulations and trained PLS and NN architectures on the simulated data to predict F and crucial properties affecting it. They found that the models performed well with more than 80% of predictions being within 2-fold error of actual values. The models performed significantly better with the addition of 44 quadratic properties to the eight independent variables indicating the importance of considering the nonlinearity of the PK relationship. While their main goal was to identify the properties essential for oral drug absorption and F for medicinal chemists, their PLS regression model performed on par with PBPK simulated estimates for CL prediction.^82^ The same group also developed specialized local quantitative structure property relationship (QSPR) models using NNs for CL prediction. Compounds within a medicinal chemistry series were used and compared to numerically fitted CL_int_ values. They found that the local QSAR models were close to their goal of within 2-fold error with only 15 to 18 measured values from PBPK simulations suggesting potential future clinical applicability.^83^ Using a similar approach, Gülave et al. predicted the ratio of unbound drug in brain extracellular fluid to plasma concentration ratio (*K*_p,uu,BBB_) using RF, SVM, kNN, PLS, and sparse PLS models, identifying RF as the top performer using both two and three-dimensional structural descriptors.^84^ Predicted parameters were then integrated into LeiCNS-PK3.0 PBPK central nervous system simulations. The authors evaluated the models against measure rat microdialysis data. Over 61% of model predicted *K*_p,uu,BBB_ values were within 3-fold error.

Schneckener and colleagues developed approaches for the prediction of rat F, AUC p.o., and AUC i.v. using *in vitro* experimental measurements, *in silico* analogs to experimental values, and chemical structure representations. They compared a traditional PBPK model with ML hybrid models using linear mapping from *in vitro* or *in silico* sources fed into a compartment model. They further included DL models in their evaluation including a DNN PBPK hybrid model, a blackbox DNN model, and a DNN PBPK model with incorporated alerts on drug exposure and bioavailability. Drug exposure in rats following i.v. administration was similarly predicted by the hybrid approaches independent of laboratory or predicted input data, whereas for oral administration *in vitro* PBPK input parameters performed significantly better demonstrating the complexity of oral drug behavior estimation. Overall, hybrid approaches were superior on regression tasks whereas the blackbox method performed slightly better in the classification task with the *in vitro* inputs unsurprisingly producing better accuracy.^20^ Handa et al. aimed to undertake the task of *C*–*t* curve prediction in the central nervous system (CNS) by building an RF model using the structural descriptors of compounds. Their three scenario approach included a positive control hybrid model with full brain compartment values derived from mouse experimental exposure data, a hybrid model with ML predicted rate parameters using compound structural descriptors, and a hybrid model using RF predicted initial values and one observed brain concentration value to which the hybrid model was refitted. In terms of accuracy, the completely fitted model dominated, while the RF model with a single concentration value followed, showing utility for sparse brain concentration data. The authors noted the limitation of the ML only predicted values being effective for compounds that show minimal deviation between plasma and brain concentration profiles.^85^ The article by Doi et al. was the only ML-mechanistic combination model applying an attention mechanism. They used AttentiveFP GNN to embed structural data for training a DNN in combination with *in vitro* and *in silico* ADME features using distinct methods. Their implicit method predicted constants for a two compartment PBPK model to predict *C*–*t* profiles and the loss function determined by comparing it to *in vivo* *C*–*t* data. The explicit method predicted PK constants which were compared to values derived from compartmental analysis and then simulated *C*–*t* profiles. The implicit method yielded more reliable PK parameter estimates and therefore better *C*–*t* predictions even when fitted with sparser *C*–*t* curve sampling. The authors attributed this to the accumulated errors from estimating individual compartment parameters in the explicit method. The explicit method is more reliant on sufficient time points coverage as parameter estimation can overlook biphasic *C*–*t* behavior driven by rapid distribution or elimination following bolus administration.^86^

Finally, two selected articles (11%) by Bräm et al. constructed NODEs for PK modeling by replacing the right side of a compartmental ODE equation with a NN aiming to capture both linear and nonlinear PK relationships. Overall, NODEs showed promising performance in various PK scenarios such as multi compartment behavior, delayed absorption, i.v. administration, and nonlinear target mediated drug disposition. The NODE models are able to simulate exposure data for subjects within the range of dosing it was trained on. This approach avoids explicit assumptions on the number of compartments of the mechanistic part of the model, however, the model does not provide information on CL or *V*_dss_ offering little explainability. The authors further present practical implementation of NODEs into pharmacometric software, and caution about potential overfitting and poor generalization on sparse PK data.^19,87^

ML-mechanistic models show promise in mitigating the effects of sparse data sets as the physics-based constraints can regularize predictions and avoid unrealistic estimations. Another notable advantage of combination architectures is the enhanced interpretability provided. Mechanistic insights are essential in early drug discovery especially for actionable interpretation of prediction results. The selected articles show a clear advantage of these hybrid approaches harnessing ML to predict input parameters directly from molecular structure while the mechanistic ODE-based components excel at propagating these values across time points and doses. It is important to note, however, that error-prone predictions may result in diminished performance of hybrid architectures due to continuous error propagation through the pipeline requiring careful mitigation.^75,86^ The overall trend in the field seems to be moving further into exploring these combination models with increasing integration of multimodal data and a variety of compound representation techniques while cautioning on overfitting and distribution model selection for compounds with no known mechanism of action.^19,75,87^

### 3.3 Barriers to Better Models

ML only and ML-mechanistic combination models demonstrate recognizable strengths for a wide array of applications in ADME/PK property prediction tasks. However, these models also present with their respective constraints, some of which both architectures share. We identified three main areas limiting the development of superior models: data (accessibility, quantity, coverage), model architecture (interpretability, inherent assumptions), and evaluation methods (benchmarking, uncertainty estimation) (Figure 5, top).

**Figure 5:**
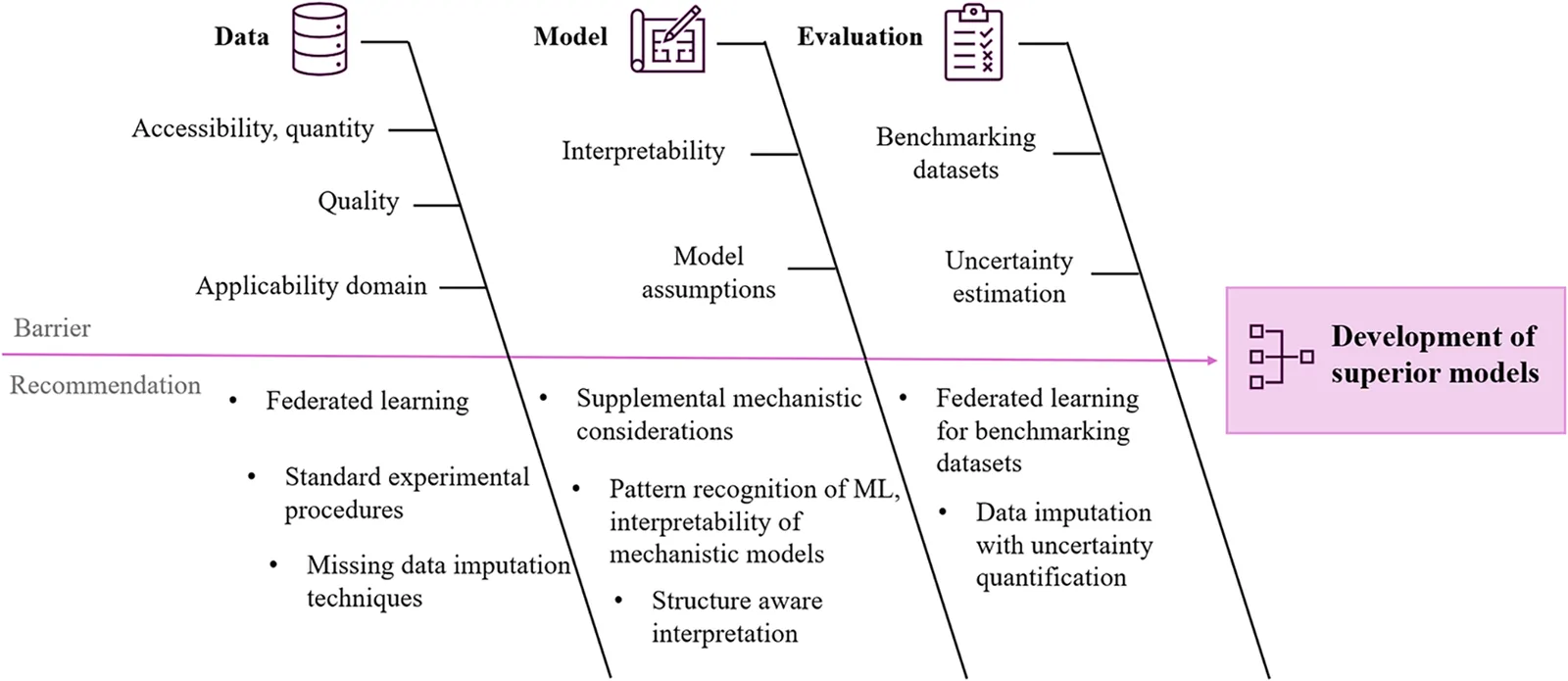
Barriers identified and recommendations summarized for the development of superior PK/ADME prediction models. Three major areas of obstacles have been identified: (1) data quantity, quality, and model applicability domain; (2) model interpretability and inherent model assumptions; and (3) limited benchmarking and uncertainty estimation techniques for evaluation (top). Some considerations include the wider adoption of federated learning approaches, effective data imputation techniques, leveraging the strengths of ML and mechanistic approaches in combination models, and balancing accuracy, interpretability, and generalizability during model development in a task dependent manner (bottom). Abbreviations: ADME—absorption, distribution, metabolism, excretion, PK—pharmacokinetic, ML—machine learning.

#### 3.3.1 Data

The most influential limitation to building powerful predictive models is the lack of sufficient, high quality data for training. Public databases offer a variety of structural, ADME/PK, pathway, and bioactivity data with nine databases identified during our analysis (Table 3), while some studies mined the literature for applicable data sets (for complete set of literature cited data sets by selected articles, see Table S1). However, there is still a lack of accessibility to more complete data sets, especially to *in vivo* PK data. Inadequate chemical space coverage, and inconsistency in experimental assays manifest in data quality problems that limit the development of prediction tools. In our analysis more than a third of the ML only methodologies used in house or proprietary data sets and prediction tools for training data consolidation (37%, 13/35). From the pool of 19 combination ML-mechanistic models, 11 (58%) used nonopen source tools and data sets bringing the total to 44% (24/54) of the overall selected records. This not only hinders academic research but compromises the ability to assess reproducibility or independently compare model performance of architectures developed in the pharmaceutical industry. The most common sources for obtaining human PK data for combination models was Elsevier’s PharmaPendium and Prosilico databases on approved drug information and PK data, each represented by four records (Table 4). The values used from these databases are not available from the respective articles due to licensing restrictions.^9,39,49,50^ Other sources of proprietary data included are summarized in Table 4.

**Table 3:** Public Databases Used by the Selected Articles and Associated Available Open Access Data

Data source public database	Selected records referencing data source	Type of data available
B3DB^91^	^72^	Blood brain barrier permeability data
BindingDB^92^	^59^	Protein-small molecule interaction data
ChEMBL^93^	^35,45,46,48–50,52,59,65,75^	Bioactivity data on drug like molecules
DrugBank^89^	^33,46,47^	Drug mechanism and target interaction data
Human Metabolome Database^94^	^33^	Data on human metabolites and associated physiological, biological, and chemical properties
KEGG^95^	^33^	Biological systems and pathway data
Metabase^96^	^42,59^	Community-based resource of biological databases
PubChem^97^	^33,36,41,46,55,58,65,75^	Bioactivity data of chemical substances
ZINC^98^	^48,54,82^	Structural data on commercially available compounds

**Table 4:** Proprietary and In House Data Sources Used by the Selected Records in This Review^a^

Data source proprietary	Selected records referencing data source	Type of data retrieved in selected article(s)
AstraZeneca	^60^	*In vivo* rat PK data
Bayer Data Warehouse	^20^	Rat oral and i.v. PK data
Boehringer Ingelheim	^61^	Physicochemical properties of compounds, *in vitro* ADME data, *in vivo* mouse, rat, and human PK data
GlaxoSmithKline (GSK)	^80,81^	ADME and human PK data
Novartis	^83^	Rat microsomal clearance data
Pfizer	^74^	Human intrinsic clearance, hepatic clearance
PharmaPendium (Elsevier)	^9,39,49,50^	Human PK data
Prosilico	^11,77–79,88^	Human PK data
Sanofi	^40^	Rat clearance, volume of distribution, and time–concentration data
Takeda	^37^	Rat clearance and microsomal unbound fraction data
Tanabe	^86^	Mouse PK data
TEIJIN Pharma database	^85^	Mouse plasma and brain PK data

a Abbreviations: ADME—absorption, distribution, metabolism, excretion, PK—pharamcokinetic, i.v.—intravenous administration.

It is important to note edge cases of accessibility such as the Mitsubishi Tanabe database that is not publicly available, but researchers may obtain deidentified PK data sets upon request.^86^ Moreover, ADMET Predictor from Simulation Plus was applied for missing data imputation, property prediction, or model building in several articles.^20,36,37,49,56,57,82,83,85^ While this software is not open source, academic licenses are available. Similarly, while DrugBank offers open source data sets (Table 3), more comprehensive data distributions are also available for academic institutions or via commercial licenses.^89^ Uniquely, the article by Chen et al. employed MeLLODDY, a framework designed for pharmaceutical companies to share data sets without revealing sensitive proprietary information such as biological targets, compound structures, or bioactivity values.^76^ MeLLODDY is an excellent example for collaborative efforts benefiting modeling research while preserving industry privacy. However, the federated learning approach is open to a limited number of institutions and companies.^90^

The limited availability of publicly accessible ADME and PK data constrains the development of robust predictive models. Furthermore, certain experimental parameters, particularly brain PK measurements are prohibitively expensive to acquire at scale and therefore remain scarce especially for novel compounds.^84,85^ Moreover, comprehensive human F data sets,^41^ or complete physicochemical sets of p*K*_a_, and partition coefficient (log *P*) values are also challenging to obtain, further impeding predictive model development.^75^ Beyond single PK end points, there are significant barriers to obtaining adequate *C*–*t* curves for CNS drugs as exposure data is often not available and carries significant costs to test in animals on a large scale. Without adequate parameter estimation using available end points, modeling *C*–*t* curves is also severely limited.^85^ Densely sampled time dependent measurements are also challenging to obtain for compounds with more novel structures. Terminal phases especially tend to have less frequent or no sampling at all, making these end ranges not well characterized by the models.^76^ Consistent with the observation by Doi and colleagues, sufficient time points are required for reliable parameter estimation, particularly for p.o. administration. On the other hand, biphasic features such as rapid distribution and elimination phases in bolus administration get flattened out by the convolution integral with insufficiently sampled data, reducing model performance in peripheral distribution prediction.^86^ Liu et al. also found that prediction accuracy suffers on sparser samples, more so with traditional NCA than their DLNCA method.^67^ These *in vivo* PK data sets would largely be available only from industrial sources following successful or failed CTs due to insufficient efficacy or toxicity concerns which are typically not released making the publicly available data sets sparser and not representative of the larger chemical space a screening library may possess. These examples all point to the urgent need to address the lack of data availability and call into question the consequences of the advancement of the PK prediction field being highly dependent on proprietary data sets that are not independently verifiable.

Data accessibility and quantity issues rapidly add up when training large predictive models. Consequently, these models become reliant on compound similarity to ones in the reference set. These reference compounds are themselves restricted in scope and may not represent structurally diverse or investigational molecules making generalization difficult.^40,59^ Additional data related limitations include class imbalance and restricted applicability domains (AD) or coverage of the training data. The AD problem manifests in limited scope of applicability of the model and lacking characterization of chemically unfavorable compounds. As highlighted by the Fagerholm group the scarcity of compounds in the extreme ends of a distribution can impair model performance, introducing complications between practical applicability and predictive accuracy especially in the screening of libraries during early drug development.^78^ The absence of end range compounds in data sets leads to poor performance for novel molecules falling within those ranges.^25,35,49,78,85^ Additionally, pharmacokinetically less favorable compounds lack experimental values for many end points. For example, compounds with low *P*_app_ are unlikely to be included in the training data set as they have not been tested *in vivo* having been deemed unfavorable for preclinical investigations.^88^ Similarly, there is also a lack of coverage on high plasma protein binding compounds.^47^ Other unfavorable ADME properties such as high molecular weight drugs would also be excluded from a model’s AD or alternatively likely to lower the accuracy of the model if included due to lower quality and sparser data points.^45,74^ This issue can be particularly problematic for classification problems, where imbalanced data sets can inflate false positive rates and produce models with limited generalizability.^9,48^

An effective model should span a broad chemical space with its AD to ensure practical utility of the model, however, expanding data sets can lead to reduced model accuracy. Conversely, models with high predictive precision frequently achieve this within a narrow AD limiting their applicability to real world drug discovery scenarios.^32^ Likewise, as previously mentioned, experimental data may only be available for optimized compounds with favorable PK properties making the extension of the AD of the model difficult. This should be taken into account especially when using sources like PharmaPendium that provides data on previously approved drugs with limited data points on less pharmacokinetically ideal compounds.^39^ Unfavorable compounds are unlikely to have *in vivo* human data on them as they would be filtered out during the drug optimization process.^49^ While models focusing only on toxicity prediction were outside the scope of this study, it is important to note that *in vivo* data availability only on optimized compounds with low adverse reaction potential also complicates the prediction of toxicity metrics of putative novel therapeutics.^74^ Due to the lack of sufficient data quantity and insufficient chemical space coverage, several researchers turned to training and validating their model on preclinical species instead, however these are not always appropriately translatable to human PK parameters and offer limited validation opportunities on human data.^20,40,73^

Experimental variability exacerbates the issue of limited data quantity by lowering the quality of the available data sets. In one case, there may be multiple measured properties reported for the same compound for the same parameter, leaving the model developer to decide which value to apply or whether to calculate the average of the values.^82^ In other cases, there may be multiple or incorrect descriptions of reported values as well as unit errors due to variable experimental conditions and reporting standards. The model developers manually correct these values and select the most appropriate data points that could potentially introduce bias.^49^ To mitigate data quantity limitations, researchers may combine data sets from several studies which further manifests the problem of varying experimental procedures and conditions.^9,69^ Contrary to this perspective, if data from a single experimental testing site is used, it may be biased toward a selected set of protocols, and the model may have poor generalizability to external data sets.^60^

#### 3.3.2 Model Architecture

Specific model architectures pose their own set of limitations in predicting PK properties that include lack of model interpretability, reliance on model assumptions, the potential for overfitting, and computational expense constraints. For instance, artificial NNs require large amounts of training data for accuracy and are typically reliant on the assumption of independence between data points,^44^ are difficult to interpret, and are prone to overfitting.^25^ Hierarchical ML models organize tasks according to groupings within the hierarchy to capture complex relationships. These models also treat each time point independently leading to disjoint *C*–*t* profile prediction.^75^ Additionally, monitoring and alleviating potential model overfitting is essential when developing various ML and DL methods to ensure practical utility.^53^ Furthermore, most prediction models, even with the addition of mechanistic components, are constrained to single dose scenarios and lack covariate evaluation in large scale applications.^19,87^ Computational expense is another important consideration that may limit these complex models. For instance, GAT networks have been reported to face batching issues when applied to larger graph data sets, making the use of more complete graphs problematic.^63^ Practical limitations of expensive computation may be mitigated using specialized data subsets for training, though this may restrict model coverage and its generalizability to unseen data.^29^

The powerful predictive capacity of ML models, especially DL methods, is frequently questioned due to lack of transparency. Model architectures with several hidden layers create difficulty in determining influential factors for individual predictions. Moreover, structure response information is critical for medicinal chemists to enable the optimization of lead compounds during drug candidate synthesis.^82^ Explainable AI is a rapidly growing field that focuses on the development of techniques to increase the interpretability of opaque ML models. The two main approaches to explainable AI are ante hoc and post hoc methods. Ante hoc methods refer to models inherently designed with clarity such as DTs, where each predicted output is easily traced back to its constituent features. Post hoc methods aim to provide insight into the relationship between input features and predicted outputs of blackbox models.^99^ NNs may offer higher accuracy, however, these are described as blackbox models lacking transparency.^80^ Several post hoc explainability methods have been explored in the field of ML and DL models, recognizing the importance of the insights drawn from knowledge accumulated and encoded in these techniques. A range of interpretability methods have been applied to these tasks. These can be broadly categorized into model agnostic approaches that can be applied to various architectures like SHapley Additive exPlanations analysis (SHAP). Perturbation-based methods such as prediction difference analysis and model-specific attribution techniques like layer-wise relevance propagation and Deep Taylor decomposition are also commonly applied. Additional approaches include testing with concept activation vectors and textual explanation frameworks for visual models.^48^

The most straightforward approach employed by three of the selected articles involves selecting various combinations and numbers of molecular descriptors and evaluating model performance for each combination to attribute feature importance similar to perturbation methods.^32,38,74^ Perturbation methods assess feature importance by shuffling model parameters such as features and assess model performance iteratively. A common implementation of this technique for preclinical and clinical data is to vary each parameter by 2-fold its normal value and evaluate model performance to determine feature importance based on fold change.^42^ Permutation techniques falling under perturbation were also used to determine feature importance by shuffling feature parameters, then assessing performance.^56,57^

SHAP is an ML technique developed for better interpretability by identifying the importance of each feature in the predictive outcome.^81^ SHAP analysis falls under the additive feature attribution methods and its model agnostic applicability has made it a popular technique for most models.^48^ There were six articles among the selected records applying SHAP.^9,41,48,58,69,81^ This technique is based on the conceptual idea of cooperative game theory.^48^ Higher positive SHAP values indicate that the feature contributes to the increase of the prediction end point value whereas negative contributes to the reduction. For instance, Wu et al. found that the SHAP values were consistent with structural preferences of compounds for CYP450 enzyme inhibition.^58^ In cases where NLP was used to encode molecular features, SHAP was still applicable for feature analysis as NLP processing does not provide importance for each token processed.^69^ Four articles applied the Boruta algorithm to select important features for training.^35,49,50,84^ The Boruta wrapper is used to calculate variable importance for the collection of features and select the most important descriptors based on RF model performance. The features are iteratively compared with model performance to optimize the model.^84^

Model-based importance analysis is another method for investigating relationships between features and predicted outcomes based on the model’s architecture. For instance, DT regression modeling with the goal of minimizing impurity (i.e., Gini index^36^) can be used for feature selection which adds transparency to the model by identifying the most relevant features and reducing those contributing mostly noise.^80^ Information gain is the metric used for selecting appropriate features that minimize impurity.^33^ This approach can also help guide medicinal chemists during rational drug design.^80^ For this goal, Chen et al. produced contour plots to visualize the relationships between predicted parameters and input features using both regression tree modeling to classify features into resulting ranges of PK end points, and SHAP analysis on the individual predictions.^80,81^ Similarly, others have investigated the relationship between descriptors among various PPB value ranges (low, medium, high) and divided the compounds into descriptor groups using classification models in addition to their NN architecture.^47^ Sensitivity coefficients can also be obtained from trained PLS models to determine feature contribution and improve general interpretability.^83^ While model-specific approaches for explainability have been popular, Iwata et al. point out that beyond their RF and XGBoost models, the same sensitivity analysis could not be compared to their DL model, making comparative evaluation cumbersome.^45^ Another approach in estimating the relative sensitivity of features in NNs, aggregates sensitivities between connecting weights from the input nodes to the output nodes through the hidden layers. This method becomes increasingly more complex with more layers. These aggregated values can be normalized as a fraction of the magnitude of the largest coefficient and then applying Fourier amplitude sensitivity testing (FAST) which automatically samples the property space providing interpretability for rational drug design. FAST has been described as a common interpretability method used in chemical, kinetic, and biological systems modeling,^83^ however, no articles in our analysis explicitly applied this method.

Local structure importance analysis appears to be more challenging to integrate into blackbox models. A distinct approach applied by Wenzel et al. introduced the response map concept for interpretability, where visualizations for local property analysis could depict property changes following fragmentation or derivatization of the input molecular structure.^52^ Iwata et al. used a linear regression model from Tensor Flow, to locally approximate the prediction of their blackbox model. This method allowed for the conversion of the contributions of the core tensor into contributions of their graph representations which in turn produces substructure-specific interpretability for the model.^45^ Another structural interpretability approach involved the visualization of substructures based on feature importance values of their individual atoms contributing to the prediction of PPB by Han et al.^47^ Furthermore, combining the power of long-range and local encoding, substructure aware cross attention mechanisms can also reliably identify chemically meaningful substructures by investigating the attention weights between global and substructure embeddings. This technique was applied using the BRICS structural representations by Li et al. and showed impressive generalizability to external data sets.^68^ Aiming to further enhance structural explainability, Jia et al. used clique descriptors, breaking molecules down into their functional groups instead of standard fingerprinting methods to allow for the identification of design rules affecting properties of interest.^72^

Uniquely, Doi et al. used integrated gradient as a feature importance analysis method.^86^ Gradient-based feature attribution methods evaluate the gradients of the model through a backward pass between the input and a baseline providing information on the relationship between the predicted output and input features.^99^ Feature importance and interpretability are inherently more transparent with combination ML-mechanistic models given the physiological parameters directly offering biological explanation from the mechanistic element.^75,76^ These combination models offer intermediate explainability, though ML predicted inputs are still difficult to adequately interpret for medicinal chemistry purposes. For example, Keefer et al. applied empirical scaling to their IVIVE model based on the second most important feature identified. This improved prediction performance, though the mechanistic link between the feature and the output was not clearly elucidated.^74^ Furthermore, NODEs have no current applicable method for explainability aside from the mechanistic assumptions of the compartment model used.^19,87^

These insights into the most recently utilized strategies reinforce the call for well suited and structurally insightful interpretability techniques for PK property prediction models while preserving predictive performance. The general goal for interpretability tools is to offer explanations with task-specific utility in a model agnostic manner, which currently is a major challenge in the field.^99^

A major obstacle arising from combination models is their dependence on the assumptions of the mechanistic architecture. For instance, a two compartment model assumption might not allow for capturing of nonlinear profiles arising from metabolic saturation or produce dual peak *C*–*t* profiles resulting from enterohepatic circulation.^86^ However, building more accurate mechanistic frameworks tailored to the data set early in drug development is often not possible due to the insufficient understanding of the underlying mechanism of action.^80^ PK predictions for bioavailability following oral administration exacerbate these problems as models typically assume liver metabolism only.^83^ The ADME complexity of oral administration proves to be more challenging to account for than predicting PK properties from i.v. bolus administration.^20,76^ Most models also lack the ability to account for nondiffusional variables such as transporter affinity,^38,50,84^ binding to nonprotein targets,^11^ or irreversible binding.^79^

Supplementary biologically relevant mechanisms may be added to improve these models. Within the realm of clearance prediction, Kosugi et al. noted that the addition of CYP enzyme involvement, hydrolysis, phase II metabolism, and extra hepatic clearance contribution (renal elimination, biliary excretion, pulmonary metabolism, plasma instability, and red blood cell partitioning) could also improve model performance and more robustly reflect the biological mechanisms of clearance.^37^ Additional considerations that may help establish more biologically accurate models are pH dependent permeability of compounds, and accounting for compounds that exhibit variable absorption often accompanied by limited reliable human data. Furthermore, taking nonfirst pass metabolism into account such as active transport and enterohepatic circulation by reabsorption can also lead to more comprehensive models.^20,55^ While the addition of these drug processing variations may improve model quality and could allow for better interpretability, model complexity and therefore computational cost will also increase rapidly.

#### 3.3.3 Evaluation Methods

Evaluating the effectiveness of complex model architectures is difficult, particularly without standardized data sets available. Several studies noted the lack of comparators and standardized data sets their models could be evaluated against.^9,25,52,57,65,88^ From our selected records, five ML only models compared their methods to mechanistic architectures. These techniques included scale up approaches, Øie-Tozer, traditional NCA, and PBPK models.^11,42,45,46,67^ The issue is multifaceted as for classification models the problem stems from imbalanced data sets with limited negative samples,^25^ or novel prediction paradigms where adequately similar models are not available for direct comparison.^65,88^ For instance, Li et al. found it difficult to compare their prediction model to others by virtue of incorporating dose as an input feature within their data set while others did not.^17^ Moreover, some models are only validated on animal PK data due to the unavailability of human *in vivo* data.^9^ Independent model evaluation is further hindered by the previously discussed limitations on data accessibility, particularly for high-performing models developed using proprietary data sets. Because these data sets are not publicly available, independent verification of reported model performance is often not possible. This lack of transparency complicates direct comparisons between models and limits the ability of external researchers aiming to reproduce published results using standardized benchmarking frameworks. Consequently, the challenge extends beyond benchmarking within individual studies and also affects systematic reviews and meta-analyses, where consistent evaluation of model performance across studies is necessary.

Interpretability methods themselves also suffer from these evaluation challenges. It is often difficult to assess how effective explainable AI-provided explanations are to analyze the relationship between features and outcomes. Blackbox models do not have a ground truth for explanations, and even if specific influential properties are highlighted, they may not align with current understanding of biomedical knowledge making validation challenging.^99^ For the above reasons, benchmarking data sets and standardized evaluation methods are urgently needed.

Quantifying prediction uncertainty is essential when evaluating model performance and improving transparency of ML models,^9^ though few studies implemented an effective strategy. The CP models generated confidence calculations over prediction intervals at specified confidence levels quantifying how different test compounds were from the training data allowing for the estimation of compound-specific uncertainty.^11,77,79,88^ Bayesian NNs were also used for integrating confidence estimation in MT PK prediction evaluating the relationships between prediction error, model uncertainty, and experimental standard deviation.^15,60^ Ensemble methods can also be leveraged to capture prediction variance.^45^ Furthermore, the Greene group produced point estimates for predictions considering experimental errors using ensemble and distribution-based approaches.^39,60^ Another approach to accessing uncertainty metrics is to obtain data sets that already have reported quality metrics associated with their data points. Jia et al. retrieved data from the B3DB database offering quality measurements for BBB penetration metrics based on the consistency of the measurements across literature reportings.^72^ Finally, Mathew and colleagues generated 90% confidence intervals using nonparametric bootstrap resampling.^42^ The studies highlight the central role of rigorous uncertainty estimation in enhancing reliability and transparency of predictive models strengthening their potential for application in drug development pipelines. However, several models rely on traditional metrics of error calculation and cross validation accuracy and fail to implement uncertainty estimation metrics.

### 3.4 Recommendations

Addressing the above-mentioned challenges is no small task. However, throughout our analysis we identified valuable suggestions to guide future prediction model development which are summarized in Figure 5, bottom.

#### 3.4.1 Data and Evaluation Related Suggestions

To address data quantity and accessibility issues, increasing federated learning collaborations could offer an invaluable resource for both the industry and academic sectors, such as the previously mentioned MeLLODDY Consortium.^76^ Federated ML is an efficient implementation of data sharing among collaborators while preserving sensitive proprietary information. These initiatives allow contributors to work on distributed data sets as one consolidated repository. Federated learning techniques extend the benefits of MT learning by prioritizing shared relationships across assays. This leads to the model being exposed to more compounds and outperforming ST models when generalizing to unseen data. Therefore, federated learning could potentially mitigate distributional heterogeneity without requiring forced data set harmonization. During the three year MeLLODDY project, in addition to reporting improvements in regression and classification performance across industry partners, applicability domains observed notable improvements. The benefits were seen across partners even for contributors providing large volumes of data, lessening the previous concerns on benefit return for federated learning.^51^ This further highlights the potential growth of individual models resulting from the application of these collaborative initiatives. Furthermore, federated learning approaches could also contribute to the construction of benchmarking data sets specific for large scale standardized model evaluation allowing for direct comparison among different architectures.^90^

Careful selection of data imputation techniques is essential to ensure a robust data set that allows for a generalizable model after training. Imputation via statistical methods, such as kNN, and multivariate imputation have been mentioned by studies but were not applied directly by the selected articles.^38,46^ On the other hand, negative sample generation methods, like synthetic minority oversampling techniques (SMOTE),^25,72^ were used to supplement or balance data sets for classification tasks. Others applied established proprietary imputation tools such as ADMEWORKS,^46,69^ and Alchemite^60^ to impute or replace PK data with *in silico* techniques. The previously mentioned ADMET Predictor remains a popular prediction tool as well with available academic licenses.^20,36,37,49,56,57,82,83,85^ However, it is important to provide adequate justification for the use and choice of missing data imputation techniques. Imputation methods may artificially amplify model performance due to data leakage raising validation concerns. Furthermore, most techniques in the selected articles offered no uncertainty quantification and consideration of biological mechanisms that may be generating the missing data in the first place. For instance, Alchemite offers standardized uncertainty quantification for predictions, however, the platform is not open source,^100^ limiting its widespread application. Conformal prediction methods offer prediction confidence quantification^11^ which could be a promising imputation technique with uncertainty estimation over simple QSAR imputation tools.

Standardized experimental procedures for the generation of high quality data sets could substantially accelerate the development of predictive models. In practice, however, data quality is inseparable from consistency which in turn depends on the adoption of standardized evaluation metrics, often the first step toward addressing data related challenges.

In response to these needs, several independent platforms have emerged to support the training and benchmarking of PK property prediction models. For example, the Therapeutics Data Commons provides curated training and testing data splits across 10 regression and 31 classification tasks in the PK domain.^101^ Similarly, ADME@NCATS offers high quality, PubChem-derived CYP enzyme inhibition and microsomal clearance data sets for model evaluation.^102^ Other resources, such as PharmaBench provides ADMET data sets including standardized SMILES representations for robust model comparisons covering microsomal clearance from human, rat, and mouse, and AMES mutagenicity tasks.^103^ MoleculeNet is also widely used for benchmarking molecular property prediction models across domains such as quantum mechanics, physical chemistry, biophysics, and physiology.^104^ Collectively, these resources represent important progress toward reproducible end point-level benchmarking; however, they only partially address the evaluation gaps identified in this review. Most current public benchmarks are better suited for individual molecular, ADME, or PK-related end point prediction than for full PK-profile evaluation. They remain limited for benchmarking workflows that require densely sampled concentration–time curves, standardized dose and route metadata, matched multispecies PK profiles, or paired *in vitro* and *in vivo* measurements.

Besides benchmarking platforms, an important consideration emphasizing the value of real-world applicability from Beckers and colleagues described training models on older studies and data sets, then using newer data sets for validation to mimic prospective application.^76^ This method is termed temporal data splitting and is the gold standard for assessing real world applicability of a model.^37,61^ Temporal splitting is a conservative evaluation that typically results in lower performance than random splitting.^37,76^ Using time splitting over random or cluster splitting techniques also assesses the model’s ability to generalize to novel chemical space that is explored in later discovery efforts.^38,76^ This approach, while critical in evaluating generalizability to a novel chemical space and new findings, may not be appropriate in cases such as on publicly available data sets and current benchmarking platforms that do not have temporally indexed structures. To improve centralized benchmarking platforms currently used splitting methods would need to move beyond currently used standard methodologies such as random and scaffold splitting in ADME data sets.

#### 3.4.2 Model Architecture Suggestions

Tackling model inherent assumptions is challenging. Incorporating supplemental biologically relevant information such as CYP involvement, phase II metabolism, extra hepatic clearance, and hydrolysis could improve model performance by representing accurate mechanisms of drug exposure.^37^ Considering pH dependent permeability as compounds travel through the digestive tract following oral administration and involving active transport and enterohepatic circulation could all be incorporated into increasing complex models.^20,55^ The addition of these drug processing variations may improve model quality and could allow for better interpretability. Model complexity and therefore computational cost will also increase rapidly highlighting the need for careful consideration of the balance between model aims and computational expense.

The ability of DL models to identify complex patterns should be harnessed to improve extrapolation to novel chemical spaces or biological systems. The increase in input modalities, such as data from several preclinical species, and addition of *in vitro* data were shown to improve model performance.^46^ Similarly, the incorporation of three-dimensional molecular information may help address persistent challenges in generalizability. Several studies in this review provide evidence that three-dimensional structural representations can improve PK prediction. For example Mamada et al. with DeepSnap generating rotational molecular images used in a CNN to learn spatial features achieved superior performance compared to molecular descriptor-based approaches.^56,57^ Gülave et al. combined conventional two and three-dimensional descriptors derived from force field minimized conformations. Although feature analysis showed that two-dimensional descriptors were highly influential, the inclusion of three-dimensional information contributed to overall performance.^84^ Similarly, Umemori et al. reported models trained on two and three-dimensional descriptors assigned substantial importance to structural features despite relatively small training data sets.^36^ Beyond explicit calculation of three-dimensional descriptors, recent advances in geometric deep learning offer a more direct approach for incorporating structural information including covalent and noncovalent molecular interactions into predictive models.^105^ Equivariance to the Euclidean group (E­(n)-equivariant) GNNs exhibiting rotational and translational symmetries^106^ and geometric transformers have also been designed to preserve structural information in molecular systems, allowing meaningful representation learning without descriptor calculation. By embedding these structural features into models directly, these DL approaches can improve extrapolation to previously unseen chemical domains and prediction of a variety of molecular properties.^107^ These architectures were largely absent from our selected studies as applications have so far been concentrated around intermolecular drug-target and protein–protein interactions^108^ however, they represent a promising future direction for PK property prediction, especially for end points that are highly dependent on stereochemistry, target interactions, and structural conformations. Considering the difficulty in accurate mathematical modeling of investigational or novel chemical entities when constructing a mechanistic model, architectures capable of learning and generalizing from molecular structural information may provide an important direction for overcoming extrapolation limitations and improve predictive models.

The optimal positioning of these architectures ultimately depends on their intended practical application achieving an ideal balance between model interpretability, generalizability, and accuracy. For instance, substructure and physicochemical feature relevance is crucial for medicinal chemists for the evaluation of synthesizability of novel compounds and optimization of lead structures. Alternatively, PK prediction prior to preclinical testing may benefit from improved accuracy with the trade-off of decreased structural transparency. Finally, to move beyond single dose scenarios and allow for the large scale application of these prediction techniques, harnessing the biological interpretability of mechanistic models and the pattern detection capability of DL approaches in combination offer the most promising avenue for superior model development.

Foundation models are increasingly being applied in molecular property prediction. Although no universal definition has been accepted, foundation models refer to architectures that are pretrained on large-scale unlabeled data sets and subsequently adapted to specific downstream tasks in a process called transfer learning. In the PK space, these models are typically pretrained on an extensive chemical data set and then fine-tuned for specific PK end points using comparatively small labeled data sets. This paradigm has shown promise for addressing several challenges identified in this review, such as the limited availability of high quality experimental PK data, poor generalizability and reduced performance on out of distribution chemical structures.^109^ Notable examples include GROVER,^110^ MolFormer,^111^ Uni-Mol3,^112^ ChemBERTa,^70^ and the more recent CheMeleon framework.^113^ These studies primarily focus on the development and evaluation of pretraining strategies, model architectures, and representation learning approaches, rather than optimization for specific PK end points.^109^ For example, GROVER emphasizes self-supervised graph-based representation learning, whereas Uni-Mol incorporated three-dimensional molecular information through a specialized tokenization framework.^112^ Foundation models tend to evaluate performance across broad benchmark suites with physicochemical, quantum mechanical, toxicity, and selected ADME end points rather than conducting detailed investigations of individual PK properties. The limited representation of foundation models in our review reflects this as this review prioritizes studies reporting models for specific PK properties. Consequently, publications focused on foundation model development and pretraining methodologies may not have met our inclusion criteria on broader filtering steps. Furthermore, this field is rapidly evolving with many influential foundation model studies initially disseminated as preprints with availability on code hosting platforms prior to peer-reviewed publication. We therefore acknowledge the limited discussion of foundation models as a limitation of the present review. Given the rapid growth of transfer learning and foundation model approaches in molecular property prediction, dedicated reviews examining their methodology and barriers would provide valuable complementary insights and may help identify emerging solutions to the data scarcity and generalizability discussed herein.

## 4 Discussion

Advances in ML and mechanistic modeling have created unprecedented opportunities for the acceleration of drug discovery and improving the prediction of PK behavior of small molecule drugs. Yet the practical value of these methods depends on the quality, completeness, and biological relevance of the data used for training and evaluation, the ability to estimate the reliability of predictions, and the architectural design choices of the model affecting performance.

Data accessibility beyond quantity and quality issues also manifests in the previously discussed differences between publicly available and proprietary PK data sets. It is difficult to distinguish model architecture effects arising from high-performing models based on proprietary data from the effects of data quality. These data sets are unavailable to the broader research community, whereas novel architectures with significant methodological advances are often evaluated on small, publicly available data sets that may contain fewer compounds, cover narrower chemical space, or may have greater assay variability. As a result, superior predictive performance may reflect the advantages of the data set size and quality rather than inherent improvements in model design. Objective assessment of methodology becomes difficult and the imbalance may overshadow true emerging architectural advancements. This disparity could shift research priorities within the field due to superior model performance attracting attention despite the inability to apply independent verification. The pharmacometric and drug development fields risk favoring approaches with underlying dense training data over truly superior modeling approaches. Greater availability to high-quality benchmark data sets with variable data splitting approaches would facilitate fairer comparisons and allow researchers to separate advances stemming from data resources from those attributable to architectural design.

Assessing model applicability through uncertainty or confidence estimations will likely take center stage in future studies. From the selected articles PKStack emerges as a strong performing model with integrated Bayesian uncertainty estimates. The authors highlight the importance of confidence scoring by showing improved performance with the removal of highly uncertain predictions. Their evaluation also urges researchers to use caution on incomplete, imbalanced or variable quality data.^9^ Given their results and the infrequent application of confidence interval estimation in PK property prediction among the selected articles, future developments should consider novel strategies to quantify uncertainty to claim clinical utility. Furthermore, improving uncertainty estimation for missing data imputation could further enhance interpretability for medicinal chemists looking to identify relevant biological relationships between structures and properties of investigational compounds. Previous evaluations of PBPK simulations relying solely on *in silico* inputs have been mostly insufficient for accurate lead compound characterization,^83^ and the use of experimental assay data consistently improved model performance.^20^ Supplementing measured data with computationally predicted samples has been the most effective approach for PK property prediction thus far, however, better oversampling techniques and transparency are required to produce more complete training data sets.

Even so, D-MPNN with Chemprop trained on assay, preclinical, and *in silico* data showed impressive performance with the authors citing potential application to human PK estimation.^60^ However, extrapolations from preclinical species have shown little success to date^20,40^ and allometric scaling has shown limited utility for their low accuracy relative to full PBPK models which require a complex set of parameters that are not available for investigational compounds. ML can support these efforts, though its use is constrained by the available training data, uncertainty calculation methods, and suffer from lack of mechanistic grounding.^80^ However, PBPK, IVIVE, allometric scaling among other mechanistic architectures can provide grounding and transparency in specific PK scenarios as well as kinetic interpretability of disease mechanisms that DL models lack, while ML and DL models can expand these models to novel chemical space and generalize better to unseen or sparse data sets as they are not restricted to mechanistic assumptions. Combination ML-PBPK approaches therefore offer the mechanistic interpretability and insight into biological processes that ML alone cannot.^80^ ML initialized IVIVE models appear to generalize well across chemical structures which is expected for mechanistic models but not guaranteed for ML models due to applicability domain constraints.^74^ These findings suggest that further investigation of combination ML-mechanistic architectures have great potential in increasing generalizability and may produce more robust multi species models, attenuating the obstacle of limited human data. Furthermore, the challenge of striking the ideal balance between predictive accuracy and interpretability may be overcome by combination models. The optimal trade-off depends on the intended application of the predictive model ranging from rapid clinical deployment to preliminary evaluation, or lead optimization where mechanistic insight into structure and PK response is critical. These hybrid models can be combined in several variations due to the versatility of ML and DL frameworks. For instance, mechanistic model input, noncompartmental analysis (NCA), or both can be estimated with ML while *C*–*t* curves simulated using PBPK models and evaluate the best performing combination. Combination ML-mechanistic models therefore offer significant advantages by leveraging the strengths of these two fundamentally different strategies.

Given the rise of these combination models, future architectural shifts expected in this field are the wider adoption of the previously mentioned foundation model and transfer learning architectures as well as further development of mixture of experts (MoE) paradigms. While we identified several ensemble models in the selected studies following various strategies such as averaging prediction probabilities to obtain final predicted probabilities^56^ or models averaging predictions from individual QSAR models.^47,72^ These techniques can help manage weaknesses or biases of individual architectures by providing complementary information. However, these are more commonly recognized as bootstrap aggregation rather than MoE paradigms. The PKStack paradigm identified in this review uses predictions from five base models as inputs into a Bayesian NN to combine them outperforming simple averaging methods^9^ which is the most closely related model to MoE paradigms as the higher level Bayesian network learns to weigh predictions of other base models. The approach reflects more of a stacked generalization, as the Bayesian network does not work directly on the input, but is providing empirical support in the direction of MoE techniques. A true MoE paradigm’s gated network would assess the input and route it to the appropriate expert models based on input content.^114^ This can be a more realistic strategy than forcing one global model to handle all outcomes with the added advantage of model-specific explainability measures, and effective scalability. Stacking and combination approaches have been extensively covered and are increasingly applied to PK prediction tasks, though true MoE models predicting these parameters have been underexplored, offering an important gap and future opportunity for research.

This review is limited by the fact that only three databases were explored. However, all papers requested for full-text screening following abstract/title screening were obtained. Another limitation is the exclusion of explicit machine learning medical subject headings from the Medline search due to the explosion of studies in recent years resulting in too much noise that is outside the scope of the current study. Additionally, purely mechanistic models were not discussed in this study, therefore the analyses do not evaluate the various standalone mechanistic modeling frameworks. Exclusion criteria also remained strict as to the precise goal of identifying barriers to scalable computational PK prediction constraining study focus.

## 5 Conclusions

While the above considerations may propel the field of computational drug discovery forward through data-driven insights, these conclusions will only be as reliable as the completeness and accuracy of the data underlying them. Lack of sufficient data quantity and varying data quality can silently impede revolutionary models through misleading results. Furthermore, inaccurate predictions with no adequate benchmarking or confidence estimation metrics can turn sophisticated prediction mechanisms useless. Continued progress therefore requires substantial efforts to improve the availability and quality of data, enhancing model interpretability, and clearly defining the intended utility of emerging predictive approaches. ML methods excel at pattern recognition and retain strengths in extrapolation to new populations and novel biological contexts, whereas mechanistic models focusing on subgroup-specific predictions and increased model transparency. Nonetheless, ML methods require improved interpretability methods and cannot yet replace the biological fidelity provided by mechanistic models. Similarly, limited extrapolation, generalization, and constrained model assumptions remain a weakness of standalone mechanistic models. Combination ML-mechanistic model building is still highly dependent on accurately capturing biological behavior which may not be possible for experimental compounds but show great potential in this field. The increased adoption of effective confidence evaluation methodologies, transfer learning-based prediction with underlying foundation models, and MoE paradigms will likely be the focal point of computational PK prediction research. The effective combination of these strategies to overcome current data, model, and evaluation barriers, requires strong collaborations between academic and industrial pharmaceutical sectors.

## Supplementary Material



## Notes

Appendix I: Key articles identified during preliminary search, Appendix II: Complete search strategy, Appendix IIa: Full text articles screened, Appendix III: Abbreviations and acronyms, and Table S1: Literature cited data sets by selected articles (PDF)
