# Supplementary material for: Gaps in AI-Driven Pharmacokinetic Property Prediction for Early Drug Development: A Scoping Review
Source: J Chem Inf Model. 2026 Aug 1;66(16):9761–83. doi: 10.1021/acs.jcim.6c01331 (PMC13544358; doi:10.1021/acs.jcim.6c01331)
Supplement: Supplementary file 1 [file ci6c01331_si_001.pdf]

Gaps in AI-driven pharmacokinetic property prediction for early drug development: a scoping review

Lucille Tomin[1], Vida Bodaghi-Namileh[1], Diane G. Schwartz[1], Ram Samudrala[1]*, Zackary Falls[1]*

[1]Department of Biomedical Informatics, Jacobs School of Medicine and Biomedical Sciences, University at Buffalo, Buffalo, NY

*Email: zmfalls@buffalo.edu, ram@compbio.org

Appendix I: Key publications

The below references were used to construct the search strategy focusing on machine learning and machine learning mechanistic combination models:

1. Kamiya Y, Handa K, Miura T, Ohori J, Kato A, Shimizu M, et al. Machine Learning Prediction of the Three Main Input Parameters of a Simplified Physiologically Based Pharmacokinetic Model Subsequently Used to Generate Time-Dependent Plasma Concentration Data in Humans after Oral Doses of 212 Disparate Chemicals. Biol Pharm Bull. 2022;45(1):124-8.
2. Li Y, Wang Z, Li Y, Du J, Gao X, Li Y, et al. A Combination of Machine Learning and PBPK Modeling Approach for Pharmacokinetics Prediction of Small Molecules in Humans. Pharm Res. 2024;41(7):1369-79.
3. Obrezanova O, Martinsson A, Whitehead T, Mahmoud S, Bender A, Miljković F, et al. Prediction of In Vivo Pharmacokinetic Parameters and Time-Exposure Curves in Rats Using Machine Learning from the Chemical Structure. Mol Pharm. 2022;19(5):1488-504.

Appendix II: Search Strategy

**Database**	**Ovid MEDLINE – Accessed 09/02/2025**	**Records up to 08/29/2025**
Search Index Number	*Search Term*	*Number of Records*
**1**	Pharmacokinetics/	9,415
**2**	Models, Biological/	365,411
**3**	Pharmaceutical Preparations/	71,583
**4**	Drug Discovery/	38,748
**5**	1 OR 3 OR 4	114,600
**6**	2 AND 5	5,837
**7**	Limit 6 to (abstracts and English language and yr="2018 - 2026")	1,026
**8**	7 NOT (peptide*.mp. or (multi adj1 molecule).tw. or (monoclonal adj1 antibod*).ti. or mAb*.ti. or PROTAC*.mp. or derivative*.mp. or nano*.ti. or screening.ti. or peptides/) not (Review or Systematic Review or Meta-analysis or Narrative Review or Clinical Trial or randomized controlled trial or controlled clinical trial).pt.	646

Key: ".tw." – text word, "/" – subject heading (MeSH), ".ti." – title, "*" - wildcard, ".mp." – multi-purpose (mp=title, book title, abstract, original title, name of substance word, subject heading word, floating sub-heading word, keyword heading word, organism supplementary concept word, protocol supplementary concept word, rare disease supplementary concept word, unique identifier, synonyms, population supplementary concept word, anatomy supplementary concept word)

**Database**	**Embase – Accessed 09/02/2025**	**Records up to 08/29/2025**
Search Index Number	*Search Term*	*Number of records*
**1**	Pharmacokinetic modeling software/exp AND machine learning/exp	260
**2**	1 AND (2018:py OR 2019:py OR 2020:py OR 2021:py OR 2022:py OR 2023:py OR 2024:py OR 2025:py) AND [embase]/lim NOT ([embase]/lim AND [medline]/lim)	102
**3**	2 AND article/it OR conference abstract/it	90

Key: ":py" – publication year, "/lim" – search limit , "/exp" – explode

**Database**	**Google Scholar – Accessed 09/02/2025**	**Records up to 08/29/2025**
Search Index Number	*Search Term*	*Number of records*
1	Pharmacokinetic modeling software AND machine learning	52
2	Publication Years: 2018-2025, Languages: English, Publication Type: Article	40

Appendix IIa: Full-text screen exclusions

Title	Authors	Year	Journal	Vol.	Issue	Pages	Exclusion Criteria	Outside scope
A Preterm Physiologically Based Pharmacokinetic Model. Part I: Physiological Parameters and Model Building	Abduljalil, K.; Pan, X.; Pansari, A.; Jamei, M.; Johnson, T. N.	2020	Clinical Pharmacokinetics	59	4	485-500	Exclusion reason: Specific population focus;	NA
Prediction of drug concentrations in milk during breastfeeding, integrating predictive algorithms within a physiologically-based pharmacokinetic model	Abduljalil, K.; Pansari, A.; Ning, J.; Jamei, M.	2021	CPT: Pharmacometrics & Systems Pharmacology	10	8	878-889	Exclusion reason: Specific population focus;	NA
Prediction of Maternal and Fetal Acyclovir, Emtricitabine, Lamivudine, and Metformin Concentrations during Pregnancy Using a Physiologically Based Pharmacokinetic Modeling Approach	Abduljalil, K.; Pansari, A.; Ning, J.; Jamei, M.	2022	Clinical Pharmacokinetics	61	5	725-748	Exclusion reason: Specific population focus;	NA
Impact of variability of in silico and in vitro octanol/water partition coefficients of compounds on the input parameters and results of simplified human physiologically based pharmacokinetic models after virtual oral administrations	Adachi, K.; Sasaki, T.; Arai, A.; Shimizu, M.; Yamazaki, H.	2024	Journal of Toxicological Sciences	49	10	459-466	Exclusion reason: No new method development;	NA
Modeled Rat Hepatic and Plasma Concentrations of Chemicals after Virtual Administrations Using Two Sets of in Silico Liver-to-Plasma Partition Coefficients	Adachi, K.; Utsumi, M.; Sato, T.; Nakano, H.; Shimizu, M.; Yamazaki, H.	2023	Biological & Pharmaceutical Bulletin	46	9	1316-1323	Exclusion reason: No new method development;	NA
A Strategy to Refine the Phenotyping Approach and Its Implementation to Predict Drug Clearance: A Physiologically	Adiwidjaja, J.; Boddy, A. V.; McLachlan, A. J.	2018	CPT: Pharmacometrics & Systems Pharmacology	7	12	798-808	Exclusion reason: Outside scope;	Mechanistic only model

Based Pharmacokinetic Simulation Study								
Physiologically-based pharmacokinetic model predictions of inter-ethnic differences in imatinib pharmacokinetics and dosing regimens	Adiwidjaja, J.; Gross, A. S.; Boddy, A. V.; McLachlan, A. J.	2022	British Journal of Clinical Pharmacology	88	4	1735-1750	Exclusion reason: Specific population focus;	NA
An ensemble learning approach for modeling the systems biology of drug-induced injury	Aguirre-Plans, J.; Pinero, J.; Souza, T.; Callegaro, G.; Kunnen, S. J.; Sanz, F.; Fernandez-Fuentes, N.; Furlong, L. I.; Guney, E.; Oliva, B.	2021	Biology Direct [Electronic Resource]	16	1	5	Exclusion reason: Outside scope;	Toxicity focus
IMI - Oral biopharmaceutics tools project - Evaluation of bottom-up PBPK prediction success part 4: Prediction accuracy and software comparisons with improved data and modelling strategies	Ahmad, A.; Pepin, X.; Aarons, L.; Wang, Y.; Darwich, A. S.; Wood, J. M.; Tannergren, C.; Karlsson, E.; Patterson, C.; Thorn, H.; Ruston, L.; Mattinson, A.; Carlert, S.; Berg, S.; Murphy, D.; Engman, H.; Laru, J.; Barker, R.; Flanagan, T.; Abrahamsson, B.; Budhdeo, S.; Franek, F.; Moir, A.; Hanisch, G.; Pathak, S. M.; Turner, D.; Jamei, M.; Brown, J.; Good, D.; Vaidhyanathan, S.; Jackson, C.;	2020	European Journal of Pharmaceutics & Biopharmaceutics	156		50-63	Exclusion reason: Outside scope;	Mechanistic only model

	Nicolas, O.; Beilles, S.; Nguefack, J. F.; Louit, G.; Henrion, L.; Ollier, C.; Boulu, L.; Xu, C.; Heimbach, T.; Ren, X.; Lin, W.; Nguyen-Trung, A. T.; Zhang, J.; He, H.; Wu, F.; Bolger, M. B.; Mullin, J. M.; van Osdol, B.; Szeto, K.; Korjamo, T.; Pappinen, S.; Tuunainen, J.; Zhu, W.; Xia, B.; Daublain, P.; Wong, S.; Varma, M. V. S.; Modi, S.; Schafer, K. J.; Schmid, K.; Lloyd, R.; Patel, A.; Tistaert, C.; Bevernage, J.; Nguyen, M. A.; Lindley, D.; Carr, R.; Rostami-Hodjegan, A.							
Prediction Accuracy of Mechanism-Based Oral Absorption Model for Dogs	Akiyama, Y.; Kimoto, T.; Mukumoto, H.; Miyake, S.; Ito, S.; Taniguchi, T.; Nomura, Y.; Matsumura, N.; Fujita, T.; Sugano, K.	2019	Journal of Pharmaceutical Sciences	108	8	2728-2736	Exclusion reason: Outside scope;	Mechanistic only model
A new model for ionizable drug dissolution in intestinal bicarbonate buffer	Al-Gousous, J.; Jalali Sohi, A.; Salehi, N.; Amidon, G. L.; Langguth, P.	2025	Journal of Pharmaceutical Sciences	114	5	103702	Exclusion reason: Outside scope;	Mechanistic only model

Computational determination of toxicity risks associated with a selection of approved drugs having demonstrated activity against COVID-19	Aminpour, M.; Delgado, W. E. M.; Wacker, S.; Noskov, S.; Houghton, M.; Tyrrell, D. L. J.; Tuszynski, J. A.	2021	BMC Pharmacology & Toxicology	22	1	61	Exclusion reason: No new method development;	NA
Time Scale Calculus: a new approach to multi-dose pharmacokinetic modeling	Arteaga-Bejarano, J. R.; Torres, S.	2024	Journal of Pharmacokinetics & Pharmacodynamics	51	6	825-839	Exclusion reason: Outside scope;	Mechanistic only model
Physiologically Based Modelling Framework for Prediction of Pulmonary Pharmacokinetics of Antimicrobial Target Site Concentrations	Aulin, L. B. S.; Tandar, S. T.; van Zijp, T.; van Ballegooie, E.; van der Graaf, P. H.; Saleh, M. A. A.; Valitalo, P.; van Hasselt, J. G. C.	2022	Clinical Pharmacokinetics	61	12	1735-1748	Exclusion reason: Outside scope;	Mechanistic only model
Breast Cancer and Exposure to Organochlorines in the CECILE Study: Associations with Plasma Levels Measured at the Time of Diagnosis and Estimated during Adolescence	Bachelet, D.; Verner, M. A.; Neri, M.; Cordina Duverger, E.; Charlier, C.; Arveux, P.; Haddad, S.; Guenel, P.	2019	International Journal of Environmental Research & Public Health [Electronic Resource]	16	2	18	Exclusion reason: Outside scope;	Single mechanism study
Prediction of the Oral Bioavailability Correlation Between Humans and Preclinical Animals	Beheshti, S.; Shayanfar, A.	2020	European Journal of Drug Metabolism & Pharmacokinetics	45	6	771-783	Exclusion reason: Outside scope;	No PK/ADME property predicted
An Accurate In Vitro Prediction of Human VD_{ss} Based on the Oie-Tozer Equation and Primary Physicochemical Descriptors. 3. Analysis and Assessment of Predictivity on a Large Dataset	Berellini, G.; Lombardo, F.	2019	Drug Metabolism & Disposition	47	12	1380-1387	Exclusion reason: No new method development;	NA
Physiologically Based Pharmacokinetic Modelling to Identify Physiological and Drug	Berton, M.; Bettonte, S.; Stader, F.;	2023	Clinical Pharmacokinetics	62	2	277-295	Exclusion reason: Specific population focus;	NA

Parameters Driving Pharmacokinetics in Obese Individuals	Battegay, M.; Marzolini, C.							
A Partial Differential Equation Approach to Inhalation Physiologically Based Pharmacokinetic Modeling	Boger, E.; Wigstrom, O.	2018	CPT: Pharmacometrics & Systems Pharmacology	7	10	638-646	Exclusion reason: Outside scope;	Mechanistic only model
Comparative Assessment of Extrapolation Methods Based on the Conventional Free Drug Hypothesis and Plasma Protein-Mediated Hepatic Uptake Theory for the Hepatic Clearance Predictions of Two Drugs Extensively Bound to Both the Albumin And Alpha-1-Acid Glycoprotein	Bteich, M.; Poulin, P.; Haddad, S.	2021	Journal of Pharmaceutical Sciences	110	3	1385-1391	Exclusion reason: Outside scope;	Single mechanism study
Validity conditions of approximations for a target-mediated drug disposition model: A novel first-order approximation and its comparison to other approximations	Byun, J. H.; Jeon, H. S.; Yun, H. Y.; Kim, J. K.	2024	PLoS Computational Biology	20	4	e1012066	Exclusion reason: Outside scope;	No PK/ADME property predicted
In-Silico Prediction of Oral Drug Bioavailability: A multi-boluses approach	Cacace, F.; Menci, M.; Papi, M.; Piemonte, V.	2021	Medical Engineering & Physics	98		140-150	Exclusion reason: Outside scope;	Mechanistic only model
Multiclassification Prediction of Enzymatic Reactions for Oxidoreductases and Hydrolases Using Reaction Fingerprints and Machine Learning Methods	Cai, Y.; Yang, H.; Li, W.; Liu, G.; Lee, P. W.; Tang, Y.	2018	Journal of Chemical Information & Modeling	58	6	1169-1181	Exclusion reason: Outside scope;	No PK/ADME property predicted
Can Population Modelling Principles be Used to Identify Key PBPK Parameters for Paediatric Clearance Predictions? An Innovative Application of Optimal Design Theory	Calvier, E. A. M.; Nguyen, T. T.; Johnson, T. N.; Rostami-Hodjegan, A.; Tibboel, D.; Krekels, E. H. J.; Knibbe, C. A. J.	2018	Pharmaceutical Research	35	11	209	Exclusion reason: Specific population focus;	NA

Steady-State Drug Exposure of Repeated IV Bolus Administration for a One Compartment Pharmacokinetic Model with Sigmoidal Hill Elimination	Cao, M.; Wu, X.; Li, J.	2024	Bulletin of Mathematical Biology	86	12	143	Exclusion reason: Outside scope;	Mechanistic only model
Exploring a Kinetic Model Approach in Biopharmaceutics: Estimating the Fraction Absorbed of Orally Administered Drugs in Humans	Chiang, P. C.; Liu, J.; Fan, P.; Wong, H.	2018	Journal of Pharmaceutical Sciences	107	7	1798-1805	Exclusion reason: Outside scope;	Mechanistic only model
Exploring Multicompartment Plug Flow-Based Model Approach in Biopharmaceutics: Impact of Stomach Setting and the Estimation of the Fraction Absorbed of Orally Administered Basic Drugs	Chiang, P. C.; Nagapudi, K.; Dolton, M. J.; Liu, J.	2020	Journal of Pharmaceutical Sciences	109	3	1261-1269	Exclusion reason: Outside scope;	Mechanistic only model
DallphinAtoM: Physiologically based pharmacokinetics software predicting human PK parameters based on physicochemical properties, in vitro and animal in vivo data	Choi, S.; Han, S.; Lee, S. J.; Lim, B.; Bae, S. H.; Han, S.; Yim, D. S.	2022	Computer Methods & Programs in Biomedicine	216		106662	Exclusion reason: Outside scope;	Mechanistic only model
Revising Pharmacokinetics of Oral Drug Absorption: II Bioavailability-Bioequivalence Considerations	Chryssafidis, P.; Tsekouras, A. A.; Macheras, P.	2021	Pharmaceutical Research	38	8	1345-1356	Exclusion reason: Outside scope;	Mechanistic only model
Re-writing Oral Pharmacokinetics Using Physiologically Based Finite Time Pharmacokinetic (PBFTPK) Models	Chryssafidis, P.; Tsekouras, A. A.; Macheras, P.	2022	Pharmaceutical Research	39	4	691-701	Exclusion reason: Outside scope;	Mechanistic only model
Relative Performance of Volume of Distribution Prediction Methods for Lipophilic Drugs with Uncertainty in LogP Value	Coutinho, A. L.; Cristofoletti, R.; Wu, F.; Al Shoyaib, A.; Dressman, J.; Polli, J. E.	2024	Pharmaceutical Research	41	6	1121-1138	Exclusion reason: Outside scope;	Single mechanism study
Development of a physiologically based pharmacokinetic (PBPK)	Cui, C.; Valerie Sia, J. E.; Tu, S.; Li, X.; Dong, Z.; Yu, Z.;	2021	British Journal of Clinical Pharmacology	87	7	2711-2722	Exclusion reason: Specific population focus;	NA

population model for Chinese elderly subjects	Yao, X.; Hatley, O.; Li, H.; Liu, D.							
Bayesian inference informed by parameter subset selection for a minimal PBPK brain model	Dadashova, K.; Smith, R. C.; Haider, M. A.; Reich, B. J.	2025	Philosophical Transactions of the Royal Society London, Series a (Mathematical, Physical & Engineering Sciences)	383	2292	20240219	Exclusion reason: Outside scope;	Mechanistic only model
A Physiologically Based Pharmacokinetic Model for Pregnant Women to Predict the Pharmacokinetics of Drugs Metabolized Via Several Enzymatic Pathways	Dallmann, A.; Ince, I.; Coboeken, K.; Eissing, T.; Hempel, G.	2018	Clinical Pharmacokinetics	57	6	749-768	Exclusion reason: Specific population focus;	NA
A novel physiologically based pharmacokinetic model of rectal absorption, evaluated and verified using clinical data on 10 rectally administered drugs	Dancik, Y.; Mittapelly, N.; Puttrevu, S. K.; Polak, S.	2023	International Journal of Pharmaceutics	643		123273	Exclusion reason: Outside scope;	Mechanistic only model
In silico drug absorption tract: An agent-based biomimetic model for human oral drug absorption	Deng, J.; Jhandey, A.; Zhu, X.; Yang, Z.; Yik, K. F. P.; Zuo, Z.; Lam, T. N.	2018	PLoS ONE [Electronic Resource]	13	8	e0203361	Exclusion reason: Outside scope;	No PK/ADME property predicted
Can membrane permeability of zwitterionic compounds be predicted by the solubility-diffusion model?	Ebert, A.; Dahley, C.	2024	European Journal of Pharmaceutical Sciences	199		106819	Exclusion reason: No new method development;	NA
Structural and functional pharmacokinetic analogs for physiologically based pharmacokinetic (PBPK) model evaluation	Ellison, C. A.	2018	Regulatory Toxicology & Pharmacology	99		61-77	Exclusion reason: Outside scope;	Mechanistic only model
Methods of Estimating Plasma Pharmacokinetics From Minimally Invasive, High Temporal Resolution Measurements of Interstitial Fluid Drug Concentrations	Erdal, M. K.; Kippin, T. E.; Hespanha, J. P.; Plaxco, K. W.	2025	Clinical and translational science	18	7	e70248	Exclusion reason: Outside scope;	Mechanistic only model

Toxic Colors: The Use of Deep Learning for Predicting Toxicity of Compounds Merely from Their Graphic Images	Fernandez, M.; Ban, F.; Woo, G.; Hsing, M.; Yamazaki, T.; LeBlanc, E.; Rennie, P. S.; Welch, W. J.; Cherkasov, A.	2018	Journal of Chemical Information & Modeling	58	8	1533-1543	Exclusion reason: Outside scope;	Toxcitiy focus
Prediction of Human Pharmacokinetics of E0703, a Novel Radioprotective Agent, Using Physiologically Based Pharmacokinetic Modeling and an Interspecies Extrapolation Approach	Ge, Y. X.; Zhang, Z.; Yan, J. Y.; Ma, Z. C.; Wang, Y. G.; Xiao, C. R.; Zhuang, X. M.; Gao, Y.	2024	International Journal of Molecular Sciences	25	5	6	Exclusion reason: Outside scope;	Mechanistic only model
Systematic evaluation of high-throughput PBK modelling strategies for the prediction of intravenous and oral pharmacokinetics in humans	Geci, R.; Gadaleta, D.; de Lomana, M. G.; Ortega-Vallbona, R.; Colombo, E.; Serrano-Candelas, E.; Paini, A.; Kuepfer, L.; Schaller, S.	2024	Archives of Toxicology	98	8	2659-2676	Exclusion reason: No new method development;	NA
How effective are ionization state-based QSPKR models at predicting pharmacokinetic parameters in humans?	Gomatam, A.; Joseph, B.; Advani, P.; Shaikh, M.; Iyer, K.; Coutinho, E.	2023	Molecular Diversity	27	4	1675-1687	Exclusion reason: No new method development;	NA
Rat PermQ: A permeability- and perfusion-based physiologically based pharmacokinetic model for improved prediction of drug concentration-time profiles in rat	Gong, Y.; Korzekwa, K.; Nagar, S.	2025	Drug Metabolism & Disposition	53	2	100033	Exclusion reason: Outside scope;	Mechanistic only model
Predicting skin permeability using the 3D-RISM-KH theory based solvation energy descriptors for a diverse class of compounds	Hinge, V. K.; Roy, D.; Kovalenko, A.	2019	Journal of Computer-Aided Molecular Design	33	6	605-611	Exclusion reason: Outside scope;	No PK/ADME property predicted
Improved Human Pharmacokinetic Prediction of Hepatically Metabolized Drugs	Horiuchi, K.; Ohnishi, S.; Matsuzaki, T.;	2018	Journal of Pharmaceutical Sciences	107	5	1443-1453	Exclusion reason: No new method development;	NA

With Species-Specific Systemic Clearance	Funaki, S.; Watanabe, A.; Mizutare, T.; Matsumoto, S.; Nezasa, K. I.; Hasegawa, H.							
Mass spectrometry methods and mathematical PK/PD model for decision tree-guided covalent drug development	Hossain, M. A.; Brahme, R. R.; Miller, B. C.; Amin, J.; de Barros, M.; Schneider, J. L.; Auclair, J. R.; Mattos, C.; Wang, Q.; Agar, N. Y. R.; Greenblatt, D. J.; Manetsch, R.; Agar, J. N.	2025	Nature Communications	16	1	1777	Exclusion reason: Outside scope;	Mechanistic only model
Prediction of the First-Pass Metabolism of a Drug After Oral Intake Based on Structural Parameters and Physicochemical Properties	Hosseini, M. A. H.; Alizadeh, A. A.; Shayanfar, A.	2024	European Journal of Drug Metabolism & Pharmacokinetics	49	4	449-465	Exclusion reason: Outside scope;	Mechanistic only model
A distributed delay approach for modeling delayed outcomes in pharmacokinetics and pharmacodynamics studies	Hu, S.; Dunlavey, M.; Guzy, S.; Teuscher, N.	2018	Journal of Pharmacokinetics & Pharmacodynamics	45	2	285-308	Exclusion reason: Outside scope;	Mechanistic only model
Novel Mechanistic PBPK Model to Predict Renal Clearance in Varying Stages of CKD by Incorporating Tubular Adaptation and Dynamic Passive Reabsorption	Huang, W.; Isoherranen, N.	2020	CPT: Pharmacometrics & Systems Pharmacology	9	10	571-583	Exclusion reason: Outside scope;	Mechanistic only model
A hybrid machine learning/pharmacokinetic approach outperforms maximum a posteriori Bayesian estimation by selectively flattening model priors	Hughes, J. H.; Keizer, R. J.	2021	CPT: Pharmacometrics & Systems Pharmacology	10	10	1150-1160	Exclusion reason: Outside scope;	No PK/ADME property predicted
Approaches to handling missing or "problematic" pharmacology data: Pharmacokinetics	Irby, D. J.; Ibrahim, M. E.; Dauki, A. M.; Badawi, M. A.;	2021	CPT: Pharmacometrics &	10	4	291-308	Exclusion reason: No new method development;	NA

	Illamola, S. M.; Chen, M.; Wang, Y.; Liu, X.; Phelps, M. A.; Mould, D. R.		Systems Pharmacology					
Determination of the Number of Tissue Groups of Kinetically Distinct Transit Time in Whole-Body Physiologically Based Pharmacokinetic (PBPK) Models II: Practical Application of Tissue Lumping Theories for Pharmacokinetics of Various Compounds	Jeong, Y. S.; Kim, M. S.; Chung, S. J.	2022	AAPS Journal	24	5	91	Exclusion reason: Outside scope;	Mechanistic only model
In silico models to predict tubular secretion or reabsorption clearance pathway using physicochemical properties and structural characteristics	Kaboudi, N.; Alizadeh, A. A.; Shayanfar, A.	2022	Xenobiotica	52	4	346-352	Exclusion reason: Outside scope;	No PK/ADME property predicted
Exploring clinically relevant dissolution specifications for oral solid dosage forms of weak acid drugs using an in silico modeling and simulation approach	Kambayashi, A.; Yomota, C.	2021	European Journal of Pharmaceutical Sciences	159		105728	Exclusion reason: Outside scope;	Single mechanism study
Physiologically Based Pharmacokinetic Models Predicting Renal and Hepatic Concentrations of Industrial Chemicals after Virtual Oral Doses in Rats	Kamiya, Y.; Otsuka, S.; Miura, T.; Yoshizawa, M.; Nakano, A.; Iwasaki, M.; Kobayashi, Y.; Shimizu, M.; Kitajima, M.; Shono, F.; Funatsu, K.; Yamazaki, H.	2020	Chemical Research in Toxicology	33	7	1736-1751	Exclusion reason: No new method development;	NA
Extrapolation of In Vivo Hepatic Clearance from In Vitro Uptake Clearance by Suspended Human Hepatocytes for Anionic Drugs with High Binding to Human Albumin: Improvement	Kim, S. J.; Lee, K. R.; Miyauchi, S.; Sugiyama, Y.	2019	Drug Metabolism & Disposition	47	2	94-103	Exclusion reason: Outside scope;	Mechanistic only model

of In Vitro-to-In Vivo Extrapolation by Considering the "Albumin-Mediated" Hepatic Uptake Mechanism on the Basis of the "Facilitated-Dissociation Model"								
Prediction of Apparent Oral Clearance of Small-Molecule Inhibitors in Pediatric Patients	Kimura, Y.; Chisaki, Y.; Saki, T.; Matsumura, C.; Motohashi, H.; Onoue, M.; Yano, Y.	2018	Journal of Pharmaceutical Sciences	107	3	949-956	Exclusion reason: Specific population focus;	NA
An Integrated Multi-Label Classifier with Chemical-Chemical Interactions for Prediction of Chemical Toxicity Effects	Liu, T.; Chen, L.; Pan, X.	2018	Combinatorial Chemistry & High Throughput Screening	21	6	403-410	Exclusion reason: Outside scope;	Toxcitiy focus
Physiologically based pharmacokinetic modeling and simulation of cannabinoids in human plasma and tissues	Liu, Y.; Sprando, R. L.	2023	Journal of Applied Toxicology	43	4	589-598	Exclusion reason: No new method development;	NA
Using Tiny-TIM Dissolution and In Silico Simulation to Accelerate Oral Product Development of a BCS Class II Compound	Luo, L.; Thakral, N. K.; Schwabe, R.; Li, L.; Chen, S.	2022	AAPS PharmSciTech	23	6	185	Exclusion reason: Outside scope;	Mechanistic only model
The computation of biomarkers in pharmacokinetics with the aid of singular perturbation methods	Ma, J.; Keener, J. P.	2018	Journal of Mathematical Biology	77	5	1407-1430	Exclusion reason: Outside scope;	Mechanistic only model
Revising Pharmacokinetics of Oral Drug Absorption: I Models Based on Biopharmaceutical/Physiological and Finite Absorption Time Concepts	Macheras, P.; Chryssafidis, P.	2020	Pharmaceutical Research	37	10	187	Exclusion reason: Outside scope;	No PK/ADME property predicted
Prediction of Tissue to Plasma Concentration Ratios of Drugs in the Rat from Experimentally Estimated Volume of Distribution: Application of Allometry	Mahmood, I.	2018	Current Drug Metabolism	19	2	155-164	Exclusion reason: Outside scope;	Mechanistic only model

Shared learning from a physiologically based pharmacokinetic modeling strategy for human pharmacokinetics prediction through retrospective analysis of Genentech compounds	Mao, J.; Ma, F.; Yu, J.; Bruyn, T.; Ning, M.; Bowman, C.; Chen, Y.	2023	Biopharmaceutics & Drug Disposition	44	4	315-334	Exclusion reason: Outside scope;	Mechanistic only model
Application of Physiologically Based Pharmacokinetic Modeling to Predict Pharmacokinetics in Healthy Japanese Subjects	Matsumoto, Y.; Cabalu, T.; Sandhu, P.; Hartmann, G.; Iwasa, T.; Yoshitsugu, H.; Gibson, C.; Uemura, N.	2019	Clinical Pharmacology & Therapeutics	105	4	1018-1030	Exclusion reason: Outside scope;	Mechanistic only model
Prediction Characteristics of Oral Absorption Simulation Software Evaluated Using Structurally Diverse Low-Solubility Drugs	Matsumura, N.; Hayashi, S.; Akiyama, Y.; Ono, A.; Funaki, S.; Tamura, N.; Kimoto, T.; Jiko, M.; Haruna, Y.; Sarashina, A.; Ishida, M.; Nishiyama, K.; Fushimi, M.; Kojima, Y.; Yoneda, K.; Nakanishi, M.; Kim, S.; Fujita, T.; Sugano, K.	2020	Journal of Pharmaceutical Sciences	109	3	1403-1416	Exclusion reason: No new method development;	NA
Development and validation of a physiology-based model for the prediction of pharmacokinetics/toxicokinetics in rabbits	Mavroudis, P. D.; Hermes, H. E.; Teutonico, D.; Preuss, T. G.; Schneckener, S.	2018	PLoS ONE [Electronic Resource]	13	3	e0194294	Exclusion reason: Outside scope;	Mechanistic only model
Successful Prediction of Human Pharmacokinetics by Improving Calculation Processes of Physiologically Based Pharmacokinetic Approach	Mayumi, K.; Ohnishi, S.; Hasegawa, H.	2019	Journal of Pharmaceutical Sciences	108	8	2718-2727	Exclusion reason: Outside scope;	Mechanistic only model
Ensemble Models Based on QuBiLS-MAS Features and	Mora, J. R.; Marrero-Ponce, Y.;	2020	Chemical Research in Toxicology	33	7	1855-1873	Exclusion reason: Outside scope;	Toxicity focus

Shallow Learning for the Prediction of Drug-Induced Liver Toxicity: Improving Deep Learning and Traditional Approaches	Garcia-Jacas, C. R.; Suarez Causado, A.							
Evaluation of the whole body physiologically based pharmacokinetic (WB-PBPK) modeling of drugs	Munir, A.; Azam, S.; Fazal, S.; Bhatti, A. I.	2018	Journal of Theoretical Biology	451		9-Jan	Exclusion reason: Outside scope;	Mechanistic only model
Evaluation of the Success of High-Throughput Physiologically Based Pharmacokinetic (HT-PBPK) Modeling Predictions to Inform Early Drug Discovery	Naga, D.; Parrott, N.; Ecker, G. F.; Olivares-Morales, A.	2022	Molecular Pharmaceutics	19	7	2203-2216	Exclusion reason: No new method development;	NA
A cross-species assessment of in silico prediction methods of steady-state volume of distribution using Simcyp simulators	Ning, M.; Fang, M.; Shah, K.; Dixit, V.; Pade, D.; Musther, H.; Neuhoff, S.	2025	Journal of Pharmaceutical Sciences	114	2	1410-1422	Exclusion reason: No new method development;	NA
Predicting reactivity to drug metabolism: beyond P450s-modelling FMOs and UGTs	Oeren, M.; Walton, P. J.; Hunt, P. A.; Ponting, D. J.; Segall, M. D.	2021	Journal of Computer-Aided Molecular Design	35	4	541-555	Exclusion reason: Outside scope;	Toxicity focus
Plasma concentrations of pemafibrate with co-administered drugs predicted by physiologically based pharmacokinetic modeling in virtual populations with renal/hepatic impairment	Ogawa, S. I.; Shimizu, M.; Yamazaki, H.	2020	Xenobiotica	50	9	1023-1031	Exclusion reason: Outside scope;	Mechanistic only model
DosePredict: A Shiny Application for Generalized Pharmacokinetics-Based Dose Predictions	Okour, M.	2020	Journal of Clinical Pharmacology	60	11	1502-1508	Exclusion reason: Outside scope;	No PK/ADME property predicted
Informatics for toxicokinetics	Padilla Mercado, G.; Cook, C.; Adkins, N.; Albrecht, L.; Cary, G.; Edwards, B.; Haggard, D. E.; Hanley, N. M.;	2025	Journal of Pharmacokinetics & Pharmacodynamics	52	3	30	Exclusion reason: Outside scope;	Toxicity focus

	Hughes, M. F.; Jarnagin, A.; Kodavanti, T. D.; Korol-Bexell, E.; Kreutz, A.; Ngo, M.; Patullo, C.; Rowan, E. G.; Huse, L. M.; Correa, V. A.; Kesic, B.; Casey, W.; Aboabdo, J.; Wolf, K.; Sayre, R.; Sharma, B.; Wall, J. T.; Yamazaki, H.; Wambaugh, J. F.; Ring, C. L.							
Development of human biotransformation QSARs and application for PBT assessment refinement	Papa, E.; Sangion, A.; Arnot, J. A.; Gramatica, P.	2018	Food & Chemical Toxicology	112		535-543	Exclusion reason: Outside scope;	No PK/ADME property predicted
Can We Predict Clinical Pharmacokinetics of Highly Lipophilic Compounds by Integration of Machine Learning or In Vitro Data into Physiologically Based Models? A Feasibility Study Based on 12 Development Compounds	Parrott, N.; Manevski, N.; Olivares-Morales, A.	2022	Molecular Pharmaceutics	19	11	3858-3868	Exclusion reason: No new method development;	NA
Naive Bayesian Models for Vero Cell Cytotoxicity	Perryman, A. L.; Patel, J. S.; Russo, R.; Singleton, E.; Connell, N.; Ekins, S.; Freundlich, J. S.	2018	Pharmaceutical Research	35	9	170	Exclusion reason: Outside scope;	Toxicity focus
A new gastro-intestinal mathematical model to study drug bioavailability	Pompa, M.; Capocelli, M.; Piemonte, V.	2019	Medical Engineering & Physics	74		106-114	Exclusion reason: Outside scope;	Mechanistic only model
First-in-Human Predictions of Hepatic Clearance for Drugs With the Well-Stirred Model: Comparative Assessment Between Models of Fraction Unbound Based Either on the	Poulin, P.	2024	Journal of Pharmaceutical Sciences	113	8	2641-2650	Exclusion reason: Outside scope;	Mechanistic only model

Free Drug Hypothesis, Albumin-Facilitated Hepatic Uptake or Dynamic Binding Kinetics								
An overview of interpretability of two models of unbound fraction that are used in combination with the well-stirred model for predicting hepatic clearance of drugs	Poulin, P.	2024	Journal of Pharmaceutical Sciences	113	11	3177-3190	Exclusion reason: Outside scope;	Mechanistic only model
Application of the Tissue Composition-Based Model to Minipig for Predicting the Volume of Distribution at Steady State and Dermis-to-Plasma Partition Coefficients of Drugs Used in the Physiologically Based Pharmacokinetics Model in Dermatology	Poulin, P.; Collet, S. H.; Atrux-Tallau, N.; Linget, J. M.; Hennequin, L.; Wilson, C. E.	2019	Journal of Pharmaceutical Sciences	108	1	603-619	Exclusion reason: Outside scope;	Mechanistic only model
A New Version of the Tissue Composition-Based Model for Improving the Mechanism-Based Prediction of Volume of Distribution at Steady-State for Neutral Drugs	Poulin, P.; Nicolas, J. M.; Bouzom, F.	2024	Journal of Pharmaceutical Sciences	113	1	118-130	Exclusion reason: Outside scope;	Mechanistic only model
Global Analysis of Models for Predicting Human Absorption: QSAR, In Vitro, and Preclinical Models	Price, E.; Kalvass, J. C.; DeGoey, D.; Hosmane, B.; Doktor, S.; Desino, K.	2021	Journal of Medicinal Chemistry	64	13	9389-9403	Exclusion reason: No new method development;	NA
A multilevel object-oriented modelling methodology for physiologically-based pharmacokinetics (PBPK): Evaluation with a semi-mechanistic pharmacokinetic model	Reig-Lopez, J.; Merino-Sanjuan, M.; Mangas-Sanjuan, V.; Prado-Velasco, M.	2020	Computer Methods & Programs in Biomedicine	189		105322	Exclusion reason: Outside scope;	Mechanistic only model
Evaluation of Generic Methods to Predict Human Pharmacokinetics Using Physiologically Based Pharmacokinetic Model for	Ren, H. C.; Sai, Y.; Chen, T.	2019	European Journal of Drug Metabolism & Pharmacokinetics	44	1	121-132	Exclusion reason: Outside scope;	Single mechanism study

Early Drug Discovery of Tyrosine Kinase Inhibitors								
A comprehensive analytical model for predicting drug absorption in the olfactory region: Application to nose-to-brain delivery	Rigaut, C.; Deruyver, L.; Goole, J.; Lambert, P.; Haut, B.	2025	International Journal of Pharmaceutics	674		125392	Exclusion reason: Outside scope;	Mechanistic only model
Mechanistic Model for the Prediction of Small-Molecule Vitreal Clearance Combining Diffusion-Limited and Permeability-Limited Clearance	Rimpela, A. K.; Cui, Y.; Sauer, A.	2021	Molecular Pharmaceutics	18	7	2703-2713	Exclusion reason: Outside scope;	Mechanistic only model
Using the LeiCNS-PK3.0 Physiologically-Based Pharmacokinetic Model to Predict Brain Extracellular Fluid Pharmacokinetics in Mice	Saleh, M. A. A.; Gulave, B.; Campagne, O.; Stewart, C. F.; Elassaiss-Schaap, J.; de Lange, E. C. M.	2023	Pharmaceutical Research	40	11	2555-2566	Exclusion reason: Outside scope;	Mechanistic only model
Modeling the Effect of Mucin Binding in the Gut on Drug Delivery	Saltzman, J.; Bendtsen, C.	2019	Bulletin of Mathematical Biology	81	9	3460-3476	Exclusion reason: Outside scope;	Single mechanism study
Investigation of Factors Affecting the Performance of in silico Volume Distribution QSAR Models for Human, Rat, Mouse, Dog & Monkey	Simeon, S.; Montanari, D.; Gleeson, M. P.	2019	Molecular Informatics	38	10	e1900059	Exclusion reason: No new method development;	NA
Validating ADME QSAR Models Using Marketed Drugs	Siramshetty, V.; Williams, J.; Nguyen, T.; Neyra, J.; Southall, N.; Mathe, E.; Xu, X.; Shah, P.	2021	SLAS discovery	26	10	1326-1336	Exclusion reason: No new method development;	NA
Evaluation of ADMET Predictor in Early Discovery Drug Metabolism and Pharmacokinetics Project Work	Sohlenius-Sternbeck, A. K.; Terelius, Y.	2022	Drug Metabolism & Disposition	50	2	95-104	Exclusion reason: No new method development;	NA
Confident application of a global human liver microsomal activity QSAR	Stalring, J.; Sohlenius-Sternbeck, A. K.;	2018	Future Medicinal Chemistry	10	13	1575-1588	Exclusion reason: Outside scope;	Mechanistic only model

	Terelius, Y.; Parkes, K.							
Computational Predictions of Nonclinical Pharmacokinetics at the Drug Design Stage	Stoyanova, R.; Katzberger, P. M.; Komissarov, L.; Khadhraoui, A.; Sach-Peltason, L.; Groebke Zbinden, K.; Schindler, T.; Manevski, N.	2023	Journal of Chemical Information & Modeling	63	2	442-458	Exclusion reason: No new method development;	NA
Physiologically based biopharmaceutics modeling of regional and colon absorption in humans	Tannergren, C.; Jadhav, H.; Eckernas, E.; Fagerberg, J.; Augustijns, P.; Sjogren, E.	2023	European Journal of Pharmaceutics & Biopharmaceutics	186		144-159	Exclusion reason: Outside scope;	Mechanistic only model
In Vitro - in Vivo Extrapolation of Hepatic Clearance in Preclinical Species	Tess, D. A.; Ryu, S.; Di, L.	2022	Pharmaceutical Research	39	7	1615-1632	Exclusion reason: Outside scope;	Mechanistic only model
Trends of in vitro pharmacological potency and in vivo pharmacokinetics parameters of modern drugs: Can the therapeutic/subtherapeutic dose be estimated from in vitro K_i and pharmacokinetic parameters?	Tsuchitani, T.; Kato, M.; Tomaru, A.; Aoki, Y.; Sugiyama, Y.	2024	Clinical and translational science	17	12	e70034	Exclusion reason: Outside scope;	Review article
Comparing Scientific Machine Learning With Population Pharmacokinetic and Classical Machine Learning Approaches for Prediction of Drug Concentrations	Valderrama, D.; Teplytska, O.; Koltermann, L. M.; Trunz, E.; Schmulenson, E.; Fritsch, A.; Jaehde, U.; Frohlich, H.	2025	CPT: Pharmacometrics & Systems Pharmacology	14	4	759-769	Exclusion reason: Outside scope;	Review article
Coupled in silico platform: Computational fluid dynamics (CFD) and physiologically-based pharmacokinetic (PBPK) modelling	Vulovic, A.; Sustersic, T.; Cvijic, S.; Ibric, S.; Filipovic, N.	2018	European Journal of Pharmaceutical Sciences	113		171-184	Exclusion reason: Outside scope;	Mechanistic only model

Predicting Pharmacokinetics in Rats Using Machine Learning: A Comparative Study Between Empirical, Compartmental, and PBPK-Based Approaches	Walter, M.; Aljayyoussi, G.; Gerner, B.; Rapp, H.; Tautermann, C. S.; Balazki, P.; Skalic, M.; Borghardt, J. M.; Humbeck, L.	2025	Clinical and translational science	18	3	e70150	Exclusion reason: Outside scope;	Review article
Nonlinear Method to Predict the Distribution of Structurally Diverse Compounds between Blood and Tissue	Wang, S.; Hu, S.; Zhang, H.	2020	Journal of Pharmaceutical Sciences	109	12	3697-3715	Exclusion reason: Outside scope;	Mechanistic only model
Human-Focused Multiparameter Optimization Scores for Rank Ordering Compounds during Early Drug Discovery: Validation of PBPK Models Based on Clinical PK Data	Wang, Y.; Wang, W.; Broccatelli, F.; Kenny, J. R.; Wright, M. R.; Sorenson, J.; Desai, P.	2025	Journal of Medicinal Chemistry	68	16	17960-17970	Exclusion reason: Outside scope;	Mechanistic only model
Segmental-Dependent Intestinal Drug Permeability: Development and Model Validation of In Silico Predictions Guided by In Vivo Permeability Values	Wolk, O.; Markovic, M.; Porat, D.; Fine-Shamir, N.; Zur, M.; Beig, A.; Dahan, A.	2019	Journal of Pharmaceutical Sciences	108	1	316-325	Exclusion reason: Outside scope;	Mechanistic only model
Applicability of the Oie-Tozer model to predict three types of distribution volume (Vd) in humans: Vd in central compartment, Vd at steady state, and Vd at beta phase	Yahata, M.; Ishii, Y.; Nakagawa, T.; Watanabe, T.; Miyawaki, I.	2020	Biopharmaceutics & Drug Disposition	41	5-Apr	151-165	Exclusion reason: Outside scope;	Mechanistic only model
Introducing the Dynamic Well-Stirred Model for Predicting Hepatic Clearance and Extraction Ratio	Yan, Z.; Ma, L.; Carione, P.; Huang, J.; Hwang, N.; Kenny, J. R.; Hop, Ceca	2024	Journal of Pharmaceutical Sciences	113	4	1094-1112	Exclusion reason: Outside scope;	Mechanistic only model
Using the Dynamic Well-Stirred Model to Extrapolate Hepatic Clearance of Organic Anion-Transporting Polypeptide Transporter Substrates without	Yan, Z.; Ma, L.; Hwang, N.; Huang, J.; Kenny, J. R.; Hop, Ceca	2024	Drug Metabolism & Disposition	52	6	548-554	Exclusion reason: Outside scope;	Mechanistic only model

Assuming Albumin-Mediated Hepatic Drug Uptake								
A pharmacokinetic-pharmacodynamic model based on the SSA-1DCNN-Attention network and the semicompartment method	Yang, J.; Li, Y.	2024	Biotechnology & Genetic Engineering Reviews	40	3	2180-2195	Exclusion reason: Outside scope;	Mechanistic only model
Global Sensitivity Analysis of the Rodgers and Rowland Model for Prediction of Tissue: Plasma Partitioning Coefficients: Assessment of the Key Physiological and Physicochemical Factors That Determine Small-Molecule Tissue Distribution	Yau, E.; Olivares-Morales, A.; Gertz, M.; Parrott, N.; Darwich, A. S.; Aarons, L.; Ogungbenro, K.	2020	AAPS Journal	22	2	41	Exclusion reason: Outside scope;	Mechanistic only model
Physiologically-Based Modeling and Interspecies Prediction of Cisplatin Pharmacokinetics	Zang, X.; Kagan, L.	2024	Journal of Pharmaceutical Sciences	113	1	158-166	Exclusion reason: Outside scope;	Mechanistic only model
Large-Scale Compartmental Model-Based Study of Preclinical Pharmacokinetic Data and Its Impact on Compound Triaging in Drug Discovery	Zhang, P. Z.; Ballard, J.; Esquivel Fagiani, F.; Smith, D.; Gibson, C.; Yu, X.	2025	Molecular Pharmaceutics	22	3	1230-1240	Exclusion reason: Outside scope;	Mechanistic only model

Appendix III: Abbreviations and acronyms

Abbreviation	Meaning
AAFE	average absolute fold error
AD	applicability domain
ADMET	absorption, distribution, metabolism, excretion, toxicity
AI	artificial intelligence
ASNN	associative neural network
AUC	area under the curve
AUMC	area under the first moment curve
BBB	blood brain barrier
BERT	bidirectional encoder representations from transformers
CatBoost	categorical boosting
CL	clearance
CL/F	oral clearance
CL_{int}	intrinsic clearance
CL_p	plasma clearance
CLR	renal clearance
C_{max}	maximum concentration
CML	conventional machine learning
CMPN	complex message passing network
CNN	convolutional neural network
CNS	central nervous system
COVID-19	coronavirus disease 2019
CP	conformal prediction
C-t	concentration time
CT	clinical trials
CYP	cytochrome P450 enzyme
DL	deep learning
DMPC	dimyristoylphosphatidylcholine
D-MPNN	deep message passing neural network
DNN	deep neural network
DT	decision tree
F	oral bioavailability
FAST	Fourier amplitude sensitivity testing
f_u	fraction of drug not bound to proteins
$f_{u,brain}$	fraction unbound drug in homogenized tissue
$f_{u,mic}$	microsomal unbound fraction
$f_{u,p}$	fraction unbound in plasma
GAT	graph attention network
GB	gradient boosting
GCNN or GCN	graph convolutional neural networks
GIN	graph isomorphism network
GMFE	geometric mean fold error
GNN	graph neural network
GP	Gaussian process
HIA	human intestinal permeability
HT	high throughput
IND	investigational new drug
i.v.	intravenous
IVIVE	*in vitro in vivo* extrapolation
kNN	k-nearest neighbor
K_p	tissue to plasma coefficient

$K_{p,uu,brain}$	unbound brain to plasma concentration ratio
LightGBM	light gradient boosting machine
LogD	octanol water distribution coefficient at pH 7.4
LSTM	long-short term memory
mAb	monoclonal antibody
MC	Monte Carlo
MCC	Matthew’s correlation coefficient
MDCK	Madin-Darby canine kidney
MeSH	medical subject heading
ML	machine learning
MLP	multilayer perceptron
MoE	mixture of experts
MRT	mean residence time
MT	multi-task
NCA	noncompartmental analysis
NLP	natural language processing
NLSD	network based label space division
NN	neural network
NODE	neural ordinary differential equation
ODE	ordinary differential equation
PBPK	physiologically based pharmacokinetic
PBrP	brain penetration related parameter
PD	pharmacodynamic
P_{eff}	human intestinal permeability coefficient
PK	pharmacokinetic
PLS	partial least squares
p.o.	per os (oral administration)
PPB	plasma protein binding
PRISMA-ScR	preferred reporting items for systematic reviews and meta-analysis extension for scoping reviews
PROTAC	proteolysis targeting chimeras
QSAR	quantitative structure activity relationship
QSPR	quantitative structure property relationship
RBF	radial basis function
RF	random forest
RMSE	root mean squared error
RNN	recurrent neural network
RRF	repeated random forest
SHAP	Shapley additive explanations analysis
SMILES	simplified molecular input line entry system
SMOTE	synthetic minority oversampling technique
SPLS	sparse partial least squares
ST	single task
SVM	support vector machine
T_{max}	time at maximum concentration
TMDD	target mediated drug disposition
$t_{1/2}$	half-life
Vdss	volume of distribution at steady state
V2/F	drug distribution after oral administration
XGBoost	extreme gradient boosting

Supplementary Table S1

**Data Source Literature based**	**Selected records referencing data source**	**Type of data**
Adenot et al. (1)	(2)	Blood brain barrier (BBB) permeability and P-gp substrate data
Benet et al. (3)	(4)	Biopharmaceutics Drug Disposition Classification System (BDDCS) classification data
Deguchi et al. (5)	(6)	*In vivo* clearance (CL) and volume of distribution at steady state (V_{dss}) in mice, rats, monkeys, dogs
Dolgikh et al. (7)	(8)	Unbound brain-to-plasma partition coefficient ($K_{p,uu,brain}$)
Gülave et al. (9)	(9)	$K_{p,uu,brain}$
Handa et al. (10)	(11)	Tissue-to-plasma partition coefficient (K_p)
Irvine et al. (12)	(13)	Apparent permeability (P_{app})
Ito et al. (14)	(4)	Reabsorption, intermediate, and secretion type classification data of compounds based on renal clearance to protein-unbound fraction glomerular filtration rate
Iwata et al. (15)	(6), (16)	Missing value imputation techniques on nonclinical data producing CL and V_{dss} data for model training
Jiang et al. (17)	(18)	Efflux transporter information
Kamiya et al. Supplementary Table S1 (19)	(19)	Rat CL and V_{dss} data, predicted tissue/plasma concentration data
Kurihara et al. (20)	(6)	PK time series data from mice, guinea pigs, rats, rabbits, monkeys, dogs, and predicted human CL and V_{dss} data
Leung et al. (21)	(6)	CL and V_{dss} data from rats, dogs, chimpanzees
Li et al. (22)	(22)	Human oral PK data
Liu et al. (23)	(8)	Brain to blood concentration ratio ($K_{p,uu,br}$)
Lombardo et al. (24), (25)	(6), (15), (26)	CL and V_{dss} data in rats, dogs, monkeys, predicted human data
Obach et al. (27)	(6), (16), (28), (29)	Human PK data
Over et al. (30)	(31)	Macrocycles structural information and permeability data
Paine et al. (32)	(6)	Predicted human renal CL, renal CL from rats and dogs
Petersson et al. (33)	(6), (28)	Predicted human V_{dss} data, rat and monkey V_{dss} data
Sayre et al. (34)	(19)	PK time series data in humans and preclinical species
Smeltz et al. (35)	(36)	*In vitro* human plasma protein binding (PPB) data
Tyzack et al. (37)	(38)	Predicted cytochrome P450 (CYP) metabolism data with estimated activation energy
Varma et al. (39)	(4)	Human renal CL data
Wang et al. (40)	(41)	BBB classification data of compounds predicted using machine learning with synthetic minority oversampling technique (SMOTE)

Zaretzki et al. (42)	(43)	XenoSite tool to predict CYP-mediated site of metabolism
Zhang et al. (44)	(8)	Rat $K_{p,uu,brain}$ data

Table S1: Literature based data sources used by selected articles documented with their referenced article and type of data.

References:

1. Adenot M, Lahana R. Blood-brain barrier permeation models: discriminating between potential CNS and non-CNS drugs including P-glycoprotein substrates. J Chem Inf Comput Sci. 2004;44(1):239-48.
2. Yuan Y, Zheng F, Zhan CG. Improved Prediction of Blood-Brain Barrier Permeability Through Machine Learning with Combined Use of Molecular Property-Based Descriptors and Fingerprints. AAPS Journal. 2018;20(3):54.
3. Benet LZ, Broccatelli F, Oprea TI. BDDCS applied to over 900 drugs. Aaps j. 2011;13(4):519-47.
4. Watanabe R, Ohashi R, Esaki T, Kawashima H, Natsume-Kitatani Y, Nagao C, et al. Development of an in silico prediction system of human renal excretion and clearance from chemical structure information incorporating fraction unbound in plasma as a descriptor. Scientific Reports. 2019;9(1):18782.
5. Deguchi T, Watanabe N, Kurihara A, Igeta K, Ikenaga H, Fusegawa K, et al. Human pharmacokinetic prediction of UDP-glucuronosyltransferase substrates with an animal scale-up approach. Drug Metab Dispos. 2011;39(5):820-9.
6. Mathew S, Tess D, Burchett W, Chang G, Woody N, Keefer C, et al. Evaluation of Prediction Accuracy for Volume of Distribution in Rat and Human Using In Vitro, In Vivo, PBPK and QSAR Methods. Journal of Pharmaceutical Sciences. 2021;110(4):1799-823.
7. Dolgikh E, Watson IA, Desai PV, Sawada GA, Morton S, Jones TM, et al. QSAR Model of Unbound Brain-to-Plasma Partition Coefficient, K(p,uu,brain): Incorporating P-glycoprotein Efflux as a Variable. J Chem Inf Model. 2016;56(11):2225-33.
8. Umemori Y, Handa K, Sakamoto S, Kageyama M, Iijima T. QSAR model to predict Kp,uu,brain with a small dataset, incorporating predicted values of related parameter. Sar and Qsar in Environmental Research. 2022;33(11):885-97.
9. Gulave B, van den Maagdenberg HW, van Boven L, van Westen GJP, de Lange ECM, van Hasselt JGC. Prediction of the Extent of Blood-Brain Barrier Transport Using Machine Learning and Integration into the LeiCNS-PK3.0 Model. Pharmaceutical Research. 2025;42(2):281-9.
10. Handa K, Sakamoto S, Kageyama M, Iijima T. Development of a 2D-QSAR Model for Tissue-to-Plasma Partition Coefficient Value with High Accuracy Using Machine Learning Method, Minimum Required Experimental Values, and Physicochemical Descriptors. European Journal of Drug Metabolism and Pharmacokinetics. 2023;48(4):341-52.
11. Handa K, Yoshimura S, Kageyama M, Iijima T. Development of Novel Methods for QSAR Modeling by Machine Learning Repeatedly: A Case Study on Drug Distribution to Each Tissue. Journal of Chemical Information & Modeling. 2024;64(9):3662-9.
12. Irvine JD, Takahashi L, Lockhart K, Cheong J, Tolan JW, Selick HE, et al. MDCK (Madin-Darby canine kidney) cells: A tool for membrane permeability screening. J Pharm Sci. 1999;88(1):28-33.
13. Patel RD, Prasanth Kumar S, Pandya HA, Solanki HA. MDCKpred: a web-tool to calculate MDCK permeability coefficient of small molecule using membrane-interaction chemical features. Toxicology Mechanisms & Methods. 2018;28(9):685-98.
14. Ito S, Ando H, Ose A, Kitamura Y, Ando T, Kusuhara H, et al. Relationship between the urinary excretion mechanisms of drugs and their physicochemical properties. J Pharm Sci. 2013;102(9):3294-301.
15. Iwata H, Matsuo T, Mamada H, Motomura T, Matsushita M, Fujiwara T, et al. Predicting Total Drug Clearance and Volumes of Distribution Using the Machine Learning-Mediated Multimodal Method through the Imputation of Various Nonclinical Data. Journal of Chemical Information & Modeling. 2022;62(17):4057-65.
16. Kwon JH, Han JY, Kim M, Kim SK, Lee DK, Kim MG. Prediction of human pharmacokinetic parameters incorporating SMILES information. Archives of Pharmacal Research. 2024;47(12):914-23.

17. Jiang D, Lei T, Wang Z, Shen C, Cao D, Hou T. ADMET evaluation in drug discovery. 20. Prediction of breast cancer resistance protein inhibition through machine learning. J Cheminform. 2020;12(1):16.
18. Abdulhameed MDM, Dey S, Xu Z, Clancy B, Desai V, Wallqvist A. MONSTROUS: a web-based chemical-transporter interaction profiler. Frontiers in Pharmacology. 2025;16.
19. Kamiya Y, Handa K, Miura T, Yanagi M, Shigeta K, Hina S, et al. In Silico Prediction of Input Parameters for Simplified Physiologically Based Pharmacokinetic Models for Estimating Plasma, Liver, and Kidney Exposures in Rats after Oral Doses of 246 Disparate Chemicals. Chemical Research in Toxicology. 2021;34(2):507-13.
20. Kurihara A, Naganuma H, Hisaoka M, Tokiwa H, Kawahara Y. Prediction of human pharmacokinetics of panipenem-betamipron, a new carbapenem, from animal data. Antimicrob Agents Chemother. 1992;36(9):1810-6.
21. Leung KH, Cohn DA, Miller RR, Doss GA, Stearns RA, Simpson RE, et al. Pharmacokinetics and disposition of L-692,429. A novel nonpeptidyl growth hormone secretagogue in preclinical species. Drug Metab Dispos. 1996;24(7):753-60.
22. Li X, Zhan M, Fang J, Liu G, Tang Y, Li W. MMPK: A Multimodal Deep Learning Framework to Predict Human Oral Pharmacokinetic Parameters. Journal of Medicinal Chemistry. 2025;68(15):16678-90.
23. Liu H, Dong K, Zhang W, Summerfield SG, Terstappen GC. Prediction of brain:blood unbound concentration ratios in CNS drug discovery employing in silico and in vitro model systems. Drug Discov Today. 2018;23(7):1357-72.
24. Lombardo F, Waters NJ, Argikar UA, Dennehy MK, Zhan J, Gunduz M, et al. Comprehensive assessment of human pharmacokinetic prediction based on in vivo animal pharmacokinetic data, part 2: clearance. J Clin Pharmacol. 2013;53(2):178-91.
25. Lombardo F, Waters NJ, Argikar UA, Dennehy MK, Zhan J, Gunduz M, et al. Comprehensive assessment of human pharmacokinetic prediction based on in vivo animal pharmacokinetic data, part 1: volume of distribution at steady state. J Clin Pharmacol. 2013;53(2):167-77.
26. Lombardo F, Bentzien J, Berellini G, Muegge I. In Silico Models of Human PK Parameters. Prediction of Volume of Distribution Using an Extensive Data Set and a Reduced Number of Parameters. Journal of Pharmaceutical Sciences. 2021;110(1):500-9.
27. Obach RS, Lombardo F, Waters NJ. Trend analysis of a database of intravenous pharmacokinetic parameters in humans for 670 drug compounds. Drug Metab Dispos. 2008;36(7):1385-405.
28. Fagerholm U, Hellberg S, Alvarsson J, Arvidsson McShane S, Spjuth O. In silico prediction of volume of distribution of drugs in man using conformal prediction performs on par with animal data-based models. Xenobiotica. 2021;51(12):1366-71.
29. Keefer CE, Chang G, Di L, Woody NA, Tess DA, Osgood SM, et al. The Comparison of Machine Learning and Mechanistic In Vitro-In Vivo Extrapolation Models for the Prediction of Human Intrinsic Clearance. Molecular Pharmaceutics. 2023;20(11):5616-30.
30. Over B, Matsson P, Tyrchan C, Artursson P, Doak BC, Foley MA, et al. Structural and conformational determinants of macrocycle cell permeability. Nat Chem Biol. 2016;12(12):1065-74.
31. Fagerholm U, Hellberg S, Alvarsson J, Spjuth O. In silico predictions of the human pharmacokinetics/toxicokinetics of 65 chemicals from various classes using conformal prediction methodology. Xenobiotica. 2022;52(2):113-8.
32. Paine SW, Ménochet K, Denton R, McGinnity DF, Riley RJ. Prediction of human renal clearance from preclinical species for a diverse set of drugs that exhibit both active secretion and net reabsorption. Drug Metab Dispos. 2011;39(6):1008-13.
33. Petersson C, Papasouliotis O, Lecomte M, Badolo L, Dolgos H. Prediction of volume of distribution in humans: analysis of eight methods and their application in drug discovery. Xenobiotica. 2020;50(3):270-9.
34. Sayre RR, Wambaugh JF, Grulke CM. Database of pharmacokinetic time-series data and parameters for 144 environmental chemicals. Scientific Data. 2020;7(1):122.
35. Smeltz M, Wambaugh JF, Wetmore BA. Plasma Protein Binding Evaluations of Per- and Polyfluoroalkyl Substances for Category-Based Toxicokinetic Assessment. Chem Res Toxicol. 2023;36(6):870-81.
36. Han Z, Xia Z, Xia J, Tetko IV, Wu S. The state-of-the-art machine learning model for plasma protein binding prediction: Computational modeling with OCHEM and experimental validation. European Journal of Pharmaceutical Sciences. 2025;204:106946.

37. Tyzack JD, Hunt PA, Segall MD. Predicting Regioselectivity and Lability of Cytochrome P450 Metabolism Using Quantum Mechanical Simulations. J Chem Inf Model. 2016;56(11):2180-93.
38. Shan X, Wang X, Li CD, Chu Y, Zhang Y, Xiong Y, et al. Prediction of CYP450 Enzyme-Substrate Selectivity Based on the Network-Based Label Space Division Method. Journal of Chemical Information & Modeling. 2019;59(11):4577-86.
39. Varma MV, Feng B, Obach RS, Troutman MD, Chupka J, Miller HR, et al. Physicochemical determinants of human renal clearance. J Med Chem. 2009;52(15):4844-52.
40. Wang Z, Yang H, Wu Z, Wang T, Li W, Tang Y, et al. In Silico Prediction of Blood-Brain Barrier Permeability of Compounds by Machine Learning and Resampling Methods. ChemMedChem. 2018;13(20):2189-201.
41. Alsenan S, Al-Turaiki I, Hafez A. A Recurrent Neural Network model to predict blood-brain barrier permeability. Computational Biology & Chemistry. 2020;89:107377.
42. Zaretzki J, Matlock M, Swamidass SJ. XenoSite: accurately predicting CYP-mediated sites of metabolism with neural networks. J Chem Inf Model. 2013;53(12):3373-83.
43. Tian S, Djoumbou-Feunang Y, Greiner R, Wishart DS. CypReact: A Software Tool for in Silico Reactant Prediction for Human Cytochrome P450 Enzymes. Journal of Chemical Information & Modeling. 2018;58(6):1282-91.
44. Zhang YY, Liu H, Summerfield SG, Luscombe CN, Sahi J. Integrating in Silico and in Vitro Approaches To Predict Drug Accessibility to the Central Nervous System. Mol Pharm. 2016;13(5):1540-50.
